# Comprehensive analysis of Ogura cytoplasmic male sterility-related genes in turnip (*Brassica rapa* ssp. *rapifera*) using RNA sequencing analysis and bioinformatics

**DOI:** 10.1371/journal.pone.0218029

**Published:** 2019-06-14

**Authors:** Sue Lin, Yingjing Miao, Shiwen Su, Jian Xu, Libo Jin, Da Sun, Renyi Peng, Li Huang, Jiashu Cao

**Affiliations:** 1 Institute of Life Sciences, Wenzhou University, Wenzhou, China; 2 Biomedicine Collaborative Innovation Center, Wenzhou, China; 3 Laboratory of Cell & Molecular Biology, Institute of Vegetable Science, Zhejiang University, Hangzhou, China; 4 Wenzhou Vocational College of Science and Technology, Wenzhou, China; Huazhong University of Science and Technology, CHINA

## Abstract

Ogura-type cytoplasmic male sterility (Ogura-CMS) has been widely used in the hybrid breeding industry for cruciferous vegetables. Turnip (*Brassica rapa* ssp. *rapifera*) is one of the most important local cruciferous vegetables in China, cultivated for its fleshy root as a flat disc. Here, morphological characteristics of an Ogura-CMS line ‘BY10-2A’ and its maintainer fertile (MF) line ‘BY10-2B’ of turnip were investigated. Ogura-CMS turnip showed a reduction in the size of the fleshy root, and had distinct defects in microspore development and tapetum degeneration during the transition from microspore mother cells to tetrads. Defective microspore production and premature tapetum degeneration during microgametogenesis resulted in short filaments and withered white anthers, leading to complete male sterility of the Ogura-CMS line. Additionally, the mechanism regulating Ogura-CMS in turnip was investigated using inflorescence transcriptome analyses of the Ogura-CMS and MF lines. The *de novo* assembly resulted in a total of 84,132 unigenes. Among them, 5,117 differentially expressed genes (DEGs) were identified, including 1,339 up- and 3,778 down-regulated genes in the Ogura-CMS line compared to the MF line. A number of functionally known members involved in anther development and microspore formation were addressed in our DEG pool, particularly genes regulating tapetum programmed cell death (PCD), and associated with pollen wall formation. Additionally, 185 novel genes were proposed to function in male organ development based on GO analyses, of which 26 DEGs were genotype-specifically expressed. Our research provides a comprehensive foundation for understanding anther development and the CMS mechanism in turnip.

## Introduction

As an important and valuable resource, male-sterile varieties are extensively exploited in crop hybrid breeding. Cytoplasmic male-sterility (CMS) is a category of male-sterility resulted from a genomic conflict between the mitochondrial and nuclear genomes, and has been extensively utilized [1]. Various types of CMS have been developed and adopted in plant breeding [2].

It has been proposed that normal microsporogenesis needs appropriate timing of tapetum degeneration and specific gene expression [3]. In CMS system, this elaborate process is complex because of the mitochondrial retrograde signaling pathway and the interaction of nuclear and organelle genomes [4–7]. Considerable variations in morphological phenotype of anther development, particularly of microspore and tapetum behaviors, arise with different nuclear backgrounds and/or cytoplasmic genotype [8]. Mostly, for example, CMS causes the premature degradation of the tapetal cells [9]. However, CMS sorghum and CMS-T maize show a persistent tapetum which likely inhibits nutrient delivery, resulting in the failure of microspore development [8,10].

Ogura-type CMS was first discovered in Japanese radish (*Raphanus sativus*) and is now widely applied in the breeding of Brassicaceae crops, such as *Brassica napus*, *B*. *juncea*, and *B*. *oleracea*, providing a classic model to probe the role of nuclear-cytoplasmic genome interactions [11]. Although interactions of specific mitochondrial gene *orf138* with different nuclear backgrounds have been reported to be responsible for Ogura-CMS, diverse floral behaviors attributed to the same cytoplasm in different species have not been fully investigated [11]. For example, anther morphology in Ogura-CMS *B*. *napus* is normal, whereas pollen development is impaired and sensitive to temperature [12–16]. However, Ogura-CMS Chinese cabbage (*B*. *rapa* ssp. *pekinensis*) shows reduced plant height and delayed flowering, has shorter filaments, and produces few and infertile pollen grains in indehiscent anthers [17]. All these differences suggest the presence of various regulatory mechanisms and/or multiple regulatory pathways in *Brassica* spp.

Recently, numerous candidate genes involved in CMS have been discovered in different species, such as onion (*Allium cepa*), cabbage (*B*. *olerace* var. *capitata*), rice (*Oryza sativa*), and pepper (*Capsicum annuum*) [7,9,18,19]. In addition, participation of miRNAs and non-coding RNAs is becoming increasingly evident in retrograde regulation of CMS [20–23]. Despite previous extensive work, no specific retrograde pathway has been reported for CMS to date and the regulatory mechanism of CMS is still largely unknown.

Exploring the molecular mechanisms underlying CMS is of great importance for improving seed yield in many crop species, especially in crucifers. As a *Brassica* root crop, turnip (*Brassica rapa* ssp. *rapifera*) has been important for human consumption for thousands of years [24]. In this study, the morphological characteristics of an Ogura-CMS line ‘BY10-2A’ and its maintainer fertile (MF) line ‘BY10-2B’ of turnip were investigated, and a detailed RNA sequencing (RNA-Seq) analysis for inflorescences in turnip was conducted. These data provide a comprehensive view on the dynamic gene expression networks and their potential roles in controlling anther development. Using pairwise comparisons, we identified 5,117 DEGs, which might respond to the mutation of the mitochondrial *ORF138* locus. Among them, 185 novel genes were proposed to function in male organ development based on GO analyses. These findings provide a comprehensive insight into the regulatory networks responsible for Ogura-CMS tapetum abnormality and pollen abortion in turnip, and demonstrate that cytoplasmic retrograde regulation is probably a principal molecular mechanism for CMS in turnip.

## Materials and methods

### Plant materials and growth conditions

Previously, the Ogura-CMS line ‘BY10-2A’ of *B*. *rapa* ssp. *rapifera* was developed by inter-specific hybridization between *B*. *rapa* ssp. *chinensis* as the Ogura-CMS cytoplasm donor and fertile *B*. *rapa* L. ssp. *rapifera*, followed by 10 recurrent generations of back-crossing. The Ogura-CMS line and its maintainer fertile (MF) line ‘BY10-2B’ were cultivated in the experimental farm of Wenzhou Vocational College of Science and Technology, Wenzhou, Zhejiang, China.

### Plant morphological analysis and floret structure observation

Plants were observed and photographed at 32, 48, 110, and 180 days after germination. The length and diameter of fleshy roots were measured at 110 and 180 days after germination. A week after the first anthesis, florets of both the Ogura-CMS and MF lines were collected. The floret structures were observed under a Leica MZ16FA stereoscopic microscope (Leica Microsystems, Wetzlar, Germany).

### Floral buds, floral organs, anthers and pollen grains morphological analysis

Ogura-CMS and MF floral buds at different anther developmental stages, and various floral organs were fixed with 2.5% glutaraldehyde in phosphate buffer (pH 7.0) overnight, and post-fixed with 1% OsO_4_ in phosphate buffer for 1 h. Subsequently, the specimens were dehydrated in a graded ethanol series (50%, 70%, 80%, 90%, 95%, 2×100%). For scanning electron microscopy, the dehydrated specimens were coated with gold-palladium in an Eiko Model IB5 ion coater (Eiko Engineering Company, Ibaraki, Japan), and photographed in a Hitachi Model TM-1000 scanning electron microscope (Tokyo, Japan) [http://dx.doi.org/10.17504/protocols.io.zz4f78w]. For semi-thin section analyses, the dehydrated specimens were embedded in Spurr resin. Semi-thin sections (1 μm) were sliced under a LKB 11800 PYRAMITOME ultramicrotome (Stockholm, Sweden) and stained with 0.5% toluidine blue. Images of anther cross-sections were obtained with a Leica DMLB fluorescence microscope (Leica Microsystems, Wetzlar, Germany) [http://dx.doi.org/10.17504/protocols.io.zz5f786]. For transmission electron microscopy (TEM), ultrathin sections (70 nm) were obtained and stained with uranyl acetate followed by alkaline lead citrate, and photographed with a Hitachi Model H-7650 transmission electron microscope (Tokyo, Japan) [http://dx.doi.org/10.17504/protocols.io.zz6f79e].

### RNA sample collection and total RNA isolation

After flowering, all floral buds of an inflorescence from the Ogura-CMS and MF lines of *B*. *rapa* ssp. *rapifera* were collected. In each case, samples were harvested and pooled from ten individual plants with transcriptome profiles representing ‘*f*’ difference, then immediately frozen in liquid nitrogen and stored at -70°C until RNA isolation. For biological repetitions, RNA was extracted from three samples using the EASYspin Plant RNA kit (Aidlab Biotechnologies Corporation, Bejing, China). RNA quality and quantity were characterized on a 1% agarose gel, and determined with a NanoPhotometer spectrophotometer (IMPLEN, CA, USA) and a Qubit RNA Assay Kit in Qubit2.0 Flurometer (Life Technologies, CA, USA). RNA integrity was assessed using the Agilent Bioanalyzer 2100 system (Agilent Technologies, CA, USA).

### Illumina sequencing and *de novo* transcriptome assembly

A total of 3 μg RNA per sample was used for library preparation using a NEBNext Ultra RNA Library Prep Kit for Illumina (NEB, USA). The library preparations were then sequenced on an Illumina Hiseq 2000 platform by the Biomarker Biotechnology Corporation (Beijing, China). Clean reads were filtered from the raw reads by removing reads containing adapter, poly-N and low-quality reads. All the downstream analyses were based on clean data with quality determined by Q20, Q30, GC-content and sequence duplication levels. Then *de novo* transcriptome assembly was accomplished using the Trinity platform [25] with min-kmer-cov set to 2 by default and all other parameters set to default. The RNA-Seq data were uploaded to the Sequence Read Archive of the National Center for Biotechnology Information (NCBI) (DOI: https://www.ncbi.nlm.nih.gov/sra/PRJNA505114; accession number: PRJNA505114).

### Annotation of differentially expressed genes

Differentially expressed genes (DEGs) were screened out using the DESeq (2010) R Package. Genes with a Benjamini-Hochberg false discovery rate (FDR) < 0.05 and a log_2_ fold change (FC) ≥ 1 or ≤ -1 in each pairwise comparison were assigned as differential expressed. DEG sequences were blast in the NCBI non-redundant protein (Nr) database, Swiss-Prot database and orthologous groups of genes (eggNOG) database, and also aligned to the Clusters of Orthologous Group (COG), Clusters of Protein homology database (KOG), and Homologous protein family (Pfam) database to predict and classify functions [26,27]. Gene ontology (GO) enrichment analysis of DEGs was performed by the topGO (2007) R packages based on a Kolmogorov-Smirnov test. Pathway analysis of DEGs was carried out to detect the important pathways, based on the database of Kyoto Encyclopedia of Genes and Genomes (KEGG). Statistical enrichment of DEGs in KEGG pathways was identified by KOBAS software using a hypergeometric test [28].

### Real-time reverse transcription polymerase chain reaction (RT-PCR) validation

Sixteen DEGs were randomly selected for validation using real-time RT-PCR. Residual RNA samples for DEG analysis were transcribed into cDNA with a HiScrip II 1^st^ Strand cDNA Synthesis Kit (Vazyme Biotech, Nanjing, China) as the template. *ACT7* was used as the normalization control [29]. Real-time RT-PCR was performed on an Applied Biosystems 7500 Real-time PCR System (ThermoFisher, MA, USA) and the relative expression levels were analyzed. Three technical repeats were performed. All primers used were listed in S1 Table.

## Results

### The Ogura-CMS turnip displays complete male sterility

**Fig 1:**
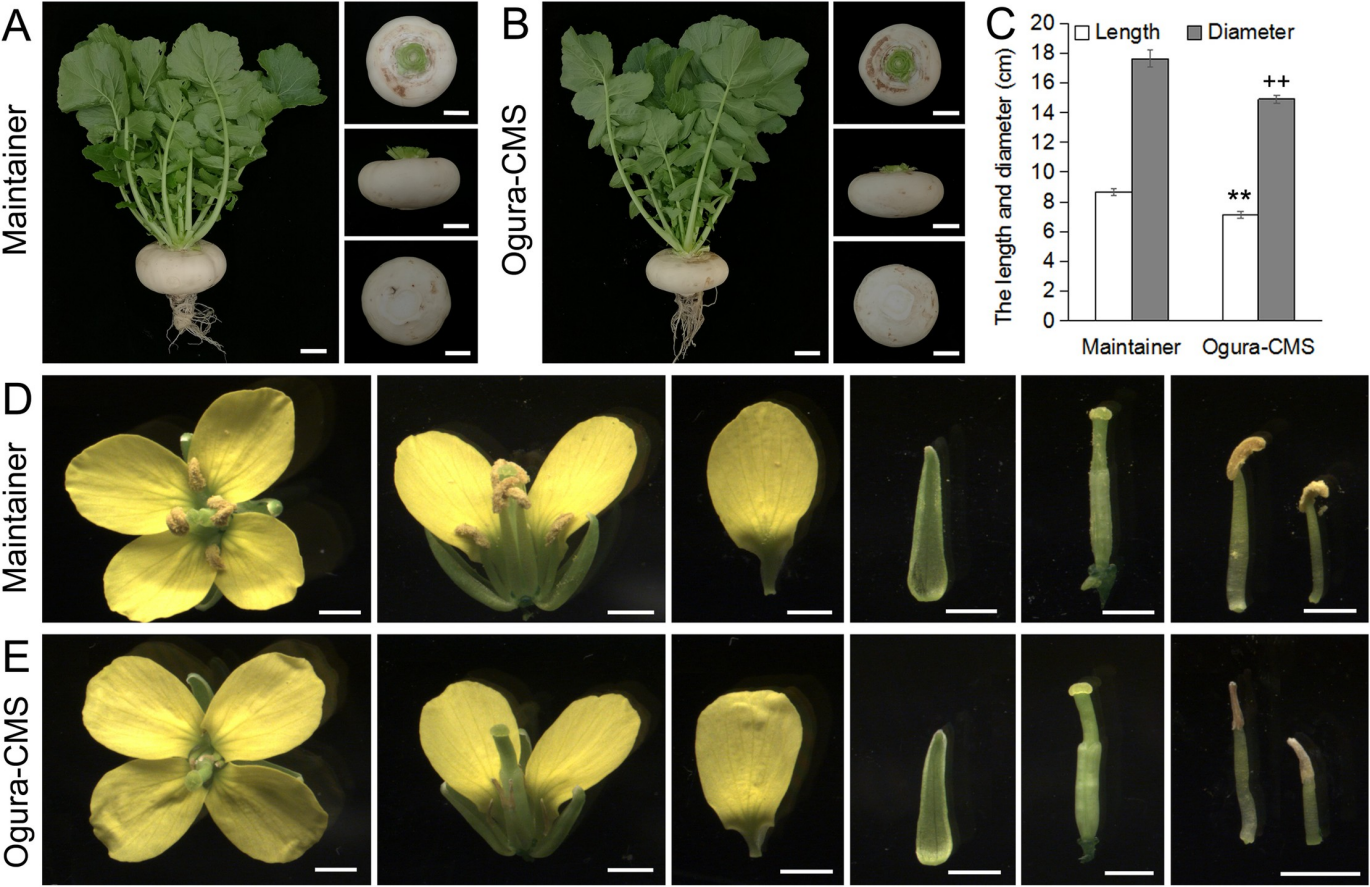
Morphological features of fleshy roots and flowers in the Ogura-CMS line and its maintainer fertile (MF) line of turnip. (A) A MF plant and its fleshy roots at 110 days after germination. (B) An Ogura-CMS plant and its fleshy roots at 110 days after germination. (C) The length and diameter of fleshy roots at 110 days after germination. The values are the mean ± SD (standard deviation). Asterisks indicate statistical significance *versus* the length of MF fleshy root (**P<0.01); crosses indicate statistical significance *versus* the diameter of MF fleshy root (^++^P<0.01). Statistical significance is determined by a one-way ANOVA test. (D) A MF floret at anthesis stage with normal floral organs. (E) An Ogura-CMS floret at anthesis stage with short filaments and withered white anthers. Bars = 5 cm in (A, B), 2 mm in (D, E).

**Fig 2:**
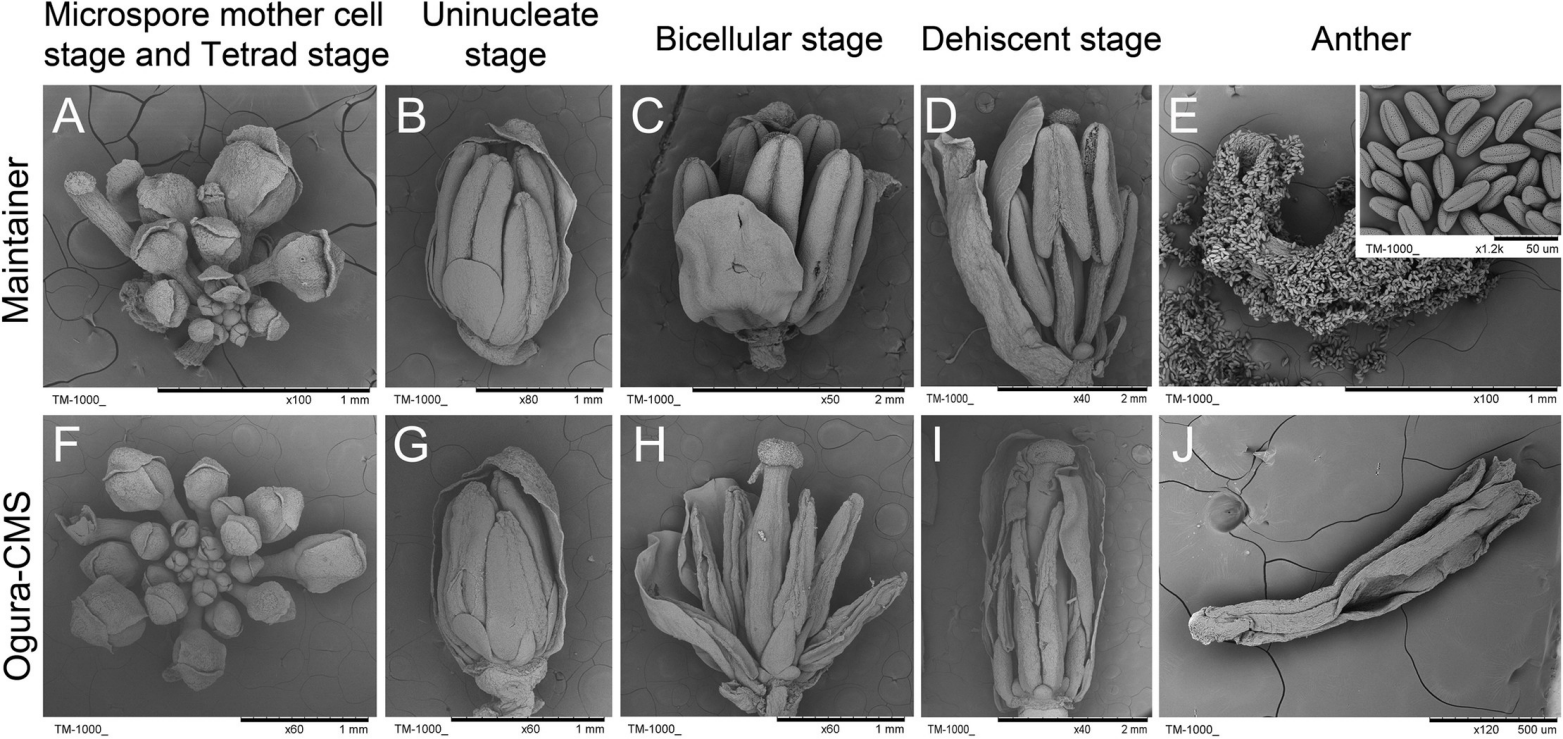
Scanning electron microscopy observation of flower development in the Ogura-CMS line and its maintainer fertile (MF) line of turnip. (A-D) Floral bud morphology in the MF line from the microspore mother cell stage to the mature pollen stage. (E) A MF dehiscent anther with normal oval pollen grains (inset shows the mature pollen grains). (F-I) Floral bud morphology in the Ogura-CMS line from the microspore mother cell stage to the mature pollen stage. (A, F) Microspore mother cell stage and tetrad stage. (B, G) Uninucleate stage. An Ogura-CMS floral bud exhibits the same morphology as a MF bud. (C, H) Bicellular stage. The withered anthers of the Ogura-CMS line are evident, compared with the lump anthers of the MF line. (D, I) Dehiscent stage. The MF anthers split open along the stomium, whereas the collapse of the Ogura-CMS anthers is evident. (J) An Ogura-CMS anther without any pollen grains. Bars = 1 mm in (A, B, E-H), 2 mm in (C, D, I), 500 μm in (J).

Despite 10 generations of back-crossings, Ogura-CMS plants showed a reduction in fleshy root size compared with its MF line (Fig 1A–1C). Furthermore, during the reproductive growth phase, the Ogura-CMS line was distinguishable from the MF line by its short filaments and withered white anthers (Fig 1D and 1E). The floral buds from the Ogura-CMS line showed the same developmental pattern as those of its MF line (Fig 2A and 2C). However, compared with the yellow and plump anthers of the MF line (Fig 2B), no pollen was observed in the mature Ogura-CMS anthers (Fig 2D). Other than pollen absence and stamen abnormality, floral organs presented normal morphologies (S1 Fig), including female gametophytes as pollinating the MF pollen grains on the Ogura-CMS stigma led to normal silique growth and full seed set (S2 Fig).

### Aberrant anther development occurs during transition from microspore mother cells to tetrads

**Fig 3:**
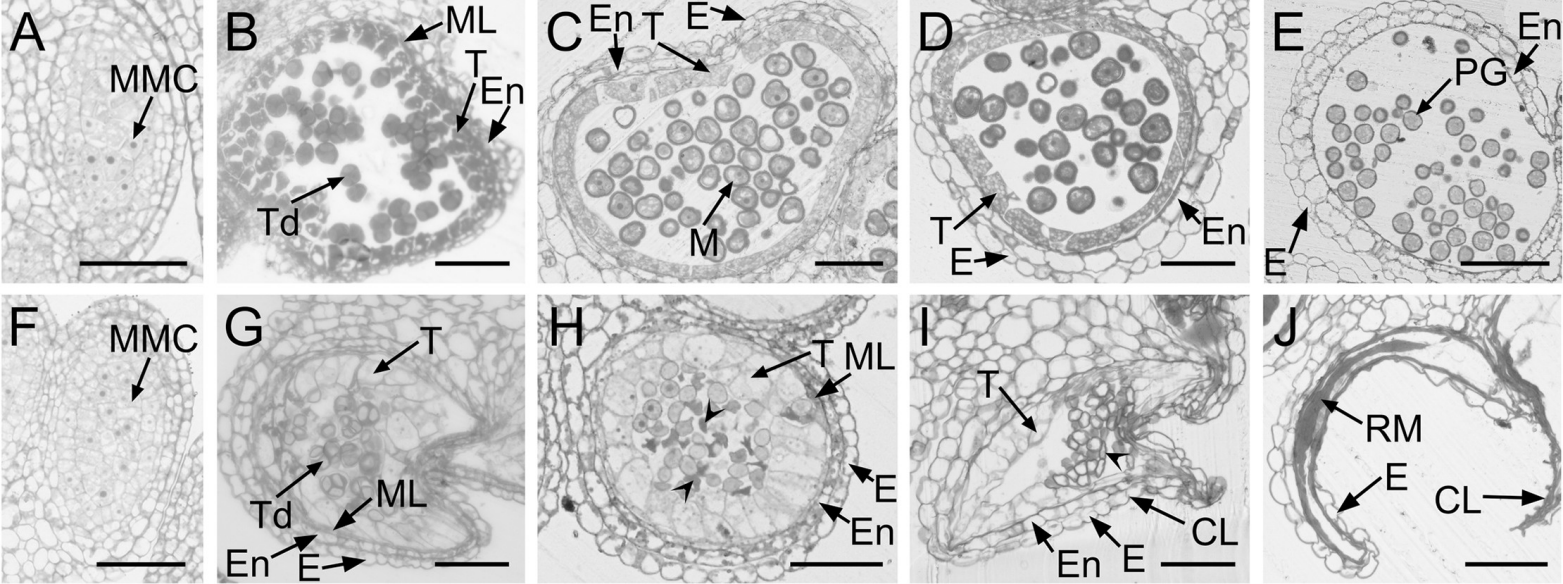
Anther and microspore development in the Ogura-CMS line and its maintainer fertile (MF) line of turnip. (A-E) Semi-thin sections of the MF anthers. (F-J) Semi-thin sections of the Ogura-CMS anthers. (A, F) Microspore mother cell stage. (B, G) Tetrad stage. The young microspores are surrounded by a callose wall, a tapetum, a middle layer, an endothecium, and an epidermis from the inside out at the tetrad stage. The tapetum in (G) swells at the center of the locule. (C, H) Uninucleate microspore stage. The middle layer persisted in (J). The aborted microspores indicated by arrowheads in (J) was surrounded by a swollen tapetal layer. (D, I) Bicellular stage. The collapse of anther locule is obvious with the aborted microspores indicated by arrowhead in (I). (E, J) Dehiscent stage. Endothecium layer is absent in the surrounding walls and remnants of the aborted microspores adhere to the inner face of the epidermis in (J). CL, collapsed locule; E, epidermis; En, endothecium; M, microspore; ML, middle layer; MMC, microspore mother cell; PG, pollen grain; RM, remnants of microspores; T, tapetum; Td, tetrads. Bars = 50 μm.

To determine the precise stage at which Ogura-CMS anther shrinkage begins, semi-thin sections of anthers from various developmental stages were prepared and further analyzed by microscopy. No differences in microspore development were observed inside the anther locules during the process of meiotic division up to the tetrad stage (Fig 3A, 3B, 3F and 3G). Each Ogura-CMS tetrad (Fig 3G) contained four microspores, similar to those of the MF line (Fig 3B), indicating that meiosis is normal in the Ogura-CMS line. Both Ogura-CMS and MF tetrads were surrounded by a tapetum, a middle layer, an endothecium, and an epidermis from the inside out at the tetrad stage (Fig 3B and 3G). However, it was noticeable that the Ogura-CMS tapetum (Fig 3G) swelled at the center of the locule, and the cytoplasm was distinguishably clear from that of the MF line (Fig 3B). After the tetrad stage, the MF middle layer degenerated, then disappeared by the uninucleate stage, and the MF anthers released microspores that developed into mature pollen grains (Fig 3C–3E). However, the collapse of the Ogura-CMS microspores started at the uninucleate microspore stage and was accompanied by extensive degeneration (Fig 3H). Moreover, the middle layer persisted at this point, together with significantly enlarged tapetum, crushing the free microspores to the locule center. At later stages of development, the collapse of the microspore was even more remarkable due to the degeneration of its entire contents (Fig 3I). Eventually, defective microspore development and early clearing of tapetal cytoplasm led to shrunken anthers with collapsed locules and remnants of pollen grains adhered to the inner face of the epidermis (Fig 3J).

**Fig 4:**
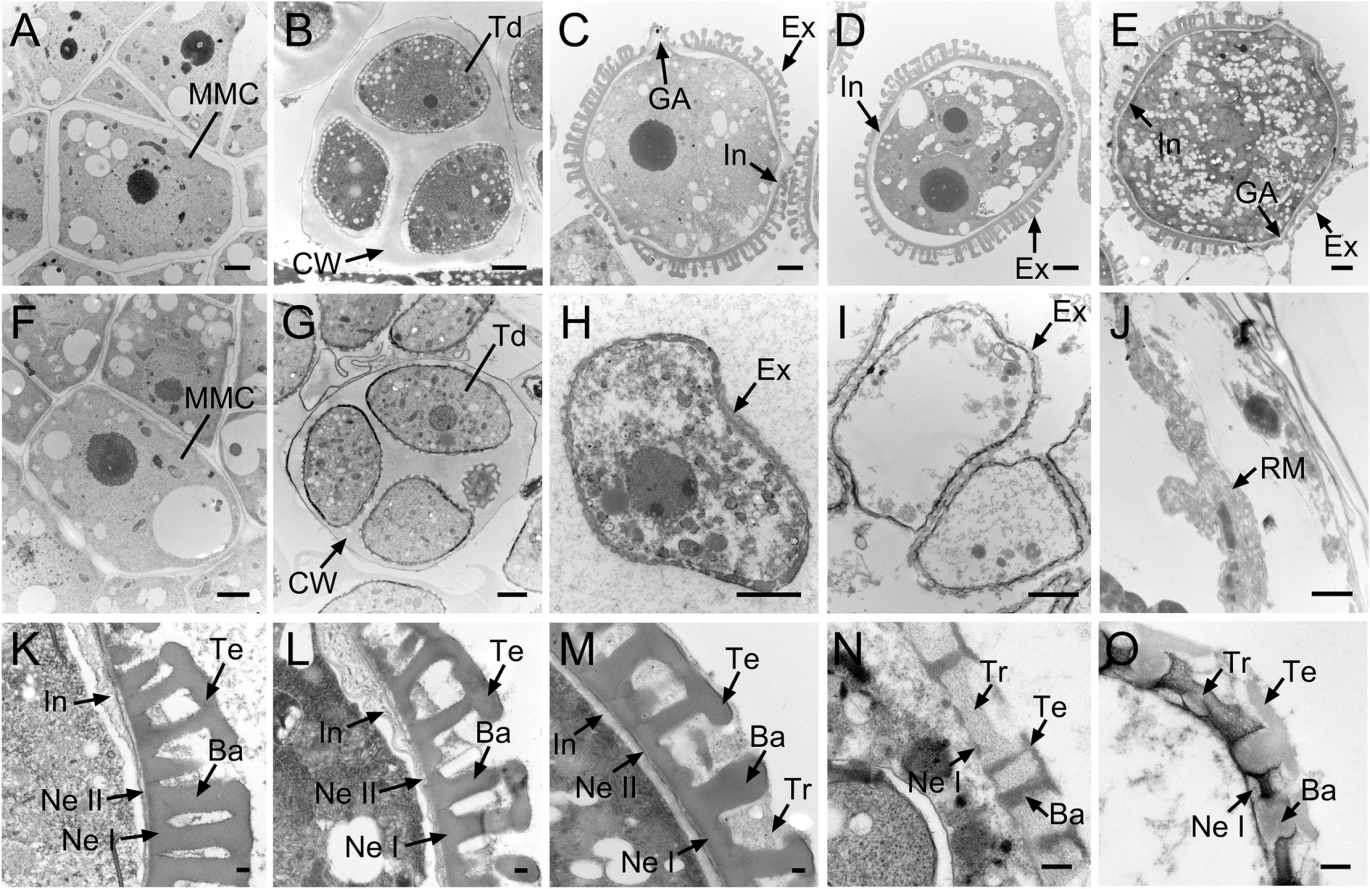
Transmission electron microscopy observation of microspore development in the Ogura-CMS line and its maintainer fertile (MF) line of turnip. (A-E) Images of microspore development in the MF line from the microspore mother cell stage to the mature pollen stage. (F-J) Images of microspore development in the Ogura-CMS line from the microspore mother cell stage to the mature pollen stage. (A, F) Microspore mother cell stage. (B, G) Tetrad stage, showing four young microspores surrounded by the callose wall. (C, H) Uninucleate microspore stage, showing the intine and the germinal apertures commenced in (C). (D, I) Bicellular stage, showing the degenerated microspores in (I). (E, J) Mature pollen stage, showing the mature pollen grain in the MF line (E) and the remnants of microspores in the Ogura-CMS line (J). (K-M) Magnified images of pollen wall in (C-E), showing the multilayered structure. (N, O) Magnified images of pollen wall in (H, I), showing the incomplete-developed exine layer and the absence of inine layer. Ba, baculum; CW, callose wall; Ex, exine; GA, germinal aperture; In, intine; M, microspore; MMC, microspore mother cell; Ne I, nexine I; Ne II, nexine II; PG, pollen grain; RM, remnants of microspores; Td, tetrads; Te, tectum; Tr, tryphine. Bars = 2 μm in (A-J), 0.2 μm in (K-O).

Abnormal Ogura-CMS microspore development was further confirmed by TEM. Ogura-CMS microspores underwent similar development to those of MF from the microspore mother cell stage to the tetrad stage (Fig 4A, 4B, 4F and 4G) and, at the uninucleate stage, were distinguishable from MF microspores (Fig 4C and 4H). At the uninucleate stage, the MF microspores had almost complete basic intine and exine structure (Fig 4C). Exine was comprised of inner nexine and outer sexine. Sexine further possessed a three-dimensional structure composed of baculae and a roof-like tectum, whereas the bilayer nexine, consisting of nexine I and nexine II, was laid down on the intine layer (Fig 4K). Subsequently, bicellular microspores were generated concurrently with the size increase of exine, and the mature exine structure was visually completed at this point (Fig 4D and 4L). Finally, the mature pollen grain was complete with tryphine (Fig 4E and 4M). However, after dissolution of the callose wall, free Ogura-CMS microspores were deformed with a thin and incomplete exine layer (Fig 4H). The nexine I was the last layer overlying the microspore plasma membrane but not the intine layer (Fig 4N). By the bicellular stage, with an empty body, pollen grains were prepared with incomplete-developed layers of exine and tryphine (Fig 4O), which developed into remnants at the dehiscent stage (Fig 4J).

**Fig 5:**
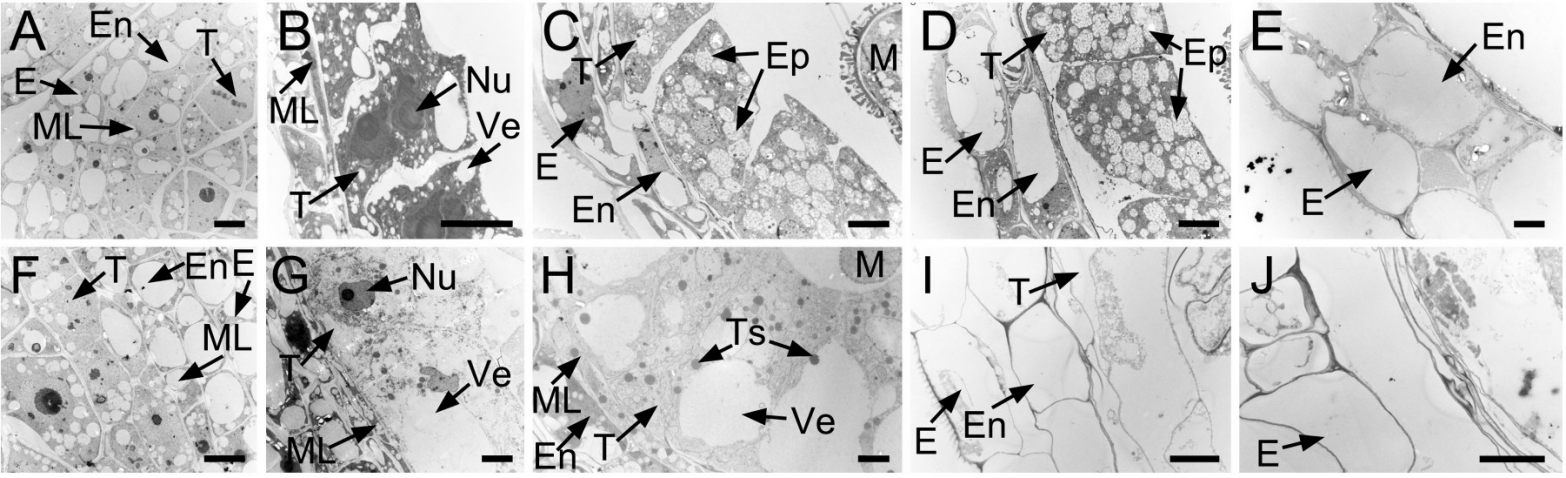
Transmission electron microscopy observation of tapetum development in the Ogura-CMS line and its maintainer fertile (MF) line of turnip. (A, F) Microspore mother cell stage. Micropores mother cells are surrounded by the tapetum, middle layer, endothecium, and epidermis from the inside out. (B, G) Tetrad stage, showing four distinctive surrounding walls and vacuolated tapetums. The tapetal cells in (G) swell to expand at the center of the locule, with larger vacuoles and a clearing cytoplasm. (C, H) Uninucleate microspore stage. Middle layer disappears and elaioplasts emerge in (C), whereas middle layer persists and tapetosomes were ubiquitous in (H). (D, I) Bicellular stage. Premature degradation of the tapetum occurs in (I), compared with integral tapetal cells with a large amount of elaioplasts in (D). (E, J) Mature pollen stage, showing the absence of the endothecium in (J). E, epidermis; En, endothecium; Ep, elaioplast; M, microspore; ML, middle layer; Nu, nuclei; T, tapetum; Ts, tapetosome; Ve, vacuole. Bars = 5 μm.

Coordinated with microspore development, visible changes occurred to the surrounding walls in the anther locules and were also observed when using TEM. No defects on Ogura-CMS surrounding walls were detected at the microspore mother cell stage (Fig 5F) compared with those of the MF line (Fig 5A). Anther primordia which were enclosed in an epidermis, differentiated inwardly into the endothecium, middle layer, tapetum, and microspore mother cells. At the tetrad stage, MF tapetal cells became mature and vacuolated, with a heterogeneous density in the cytoplasm (Fig 5B). However, the anther pattern was significantly altered in Ogura-CMS tapetum at this stage. Ogura-CMS tapetal cells enlarged and swelled to expand to the center of the locules, with larger vacuoles and a clearing cytoplasm (Fig 5G). When the callose wall totally dissolved and free individual microspores released into the anther locules, a large number of elaioplasts emerged in the MF tapetum, and remnants of the middle layer were absent in the MF anther (Fig 5C), but still clearly present in the Ogura-CMS anther (Fig 5H). In addition, Ogura-CMS tapetal cells were full of shedding materials such as tapetosomes and significantly swelled, crushing the free microspores to the locule center (Fig 5H). This premature degradation of the tapetum was obvious, which fulfilled the programmed cell death ahead of schedule. All that remained of the Ogura-CMS tapetum was an almost empty shell at the bicellular stage (Fig 5I), but integral tapetal cells with a large amount of elaioplasts were still observed in the MF anther (Fig 5D). Moreover, the endothecium also appeared abnormal in the Ogura-CMS anther, devoid of any content in the cytoplasm at the bicellular stage (Fig 5I), and had disappeared completely at the mature pollen stage (Fig 5J).

Overall, the Ogura-CMS anthers showed two distinct defects that occurred during the transition from microspore mother cells to tetrads: the failure of microspore development and the swollen tapetum layer. Defective microspore production and premature tapetum degeneration during microgametogenesis led to complete male sterility of the Ogura-CMS line.

### RNA-Seq analysis on inflorescences of the Ogura-CMS line and its maintainer fertile line in turnip

**Fig 6:**
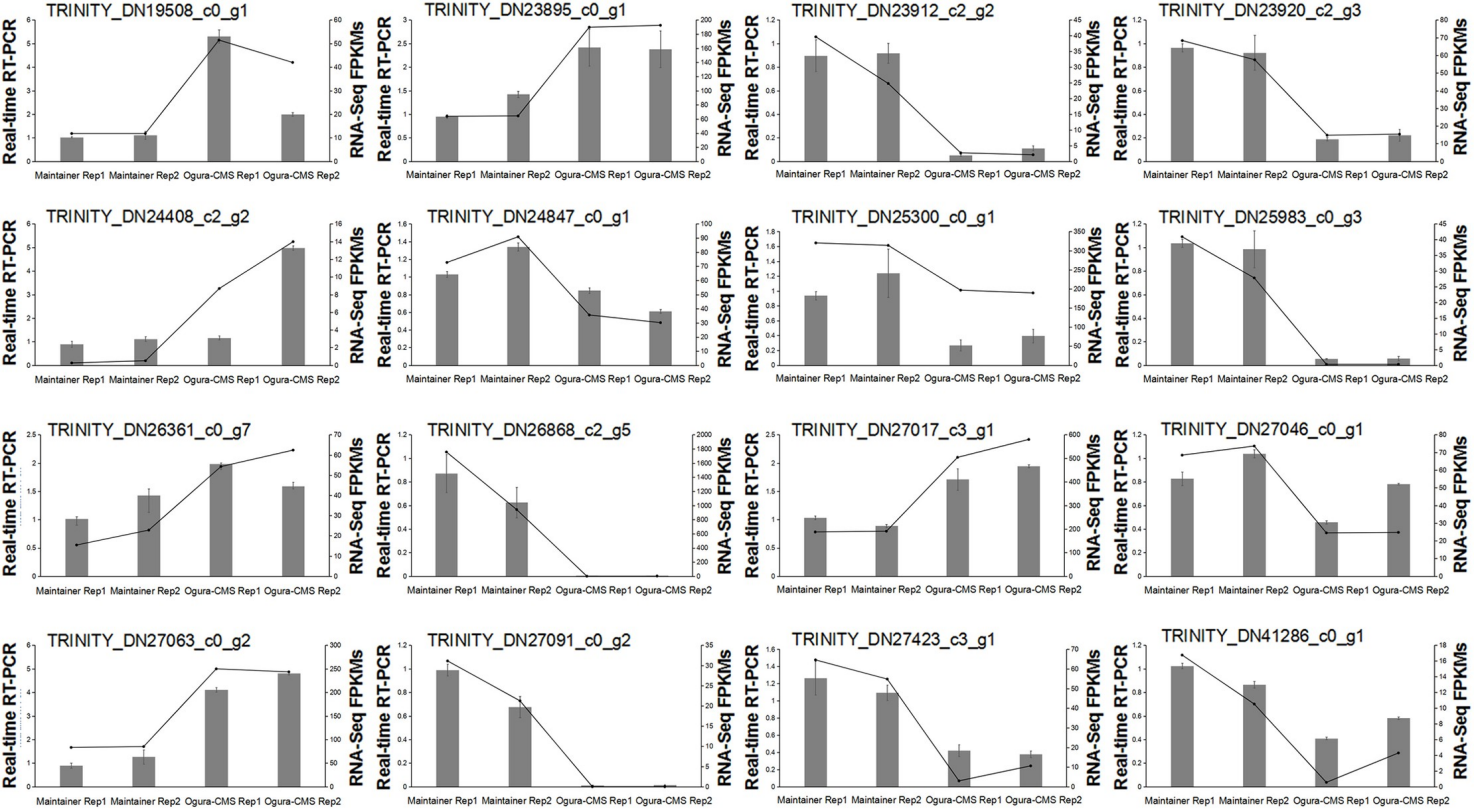
Experimental validation of the quality of the RNA-Seq data by real-time RT-PCR. The columns indicate the relative RNA levels of selected differentially expressed genes (DEGs) identified between the Ogura-CMS inflorescences and its maintainer inflorescences. The lines show the FPKM expression data of RNA-Seq.

To explore the molecular basis for the morphological differences in anther and microspore development described above, RNA-Seq analyses were conducted to generate transcriptome profiles of the whole inflorescences from the Ogura-CMS and MF lines. RNA-Seq analysis was performed with three biological replicates for each. After removing low-quality reads, an average of 24.2 × 10^6^ clean reads per library were generated (S2 Table). The *de novo* assembly resulted in a total of 84,132 unigenes (S3 Fig and S3 Table), which were proposed to be expressed during floral bud development in turnip. To verify the quality of the RNA-Seq data, real-time RT-PCR analysis was conducted on 16 randomly selected genes (Fig 6). The strong correlation between the RNA-Seq and real-time RT-PCR results indicated high reliability of our transcriptomic profiling data.

**Fig 7:**
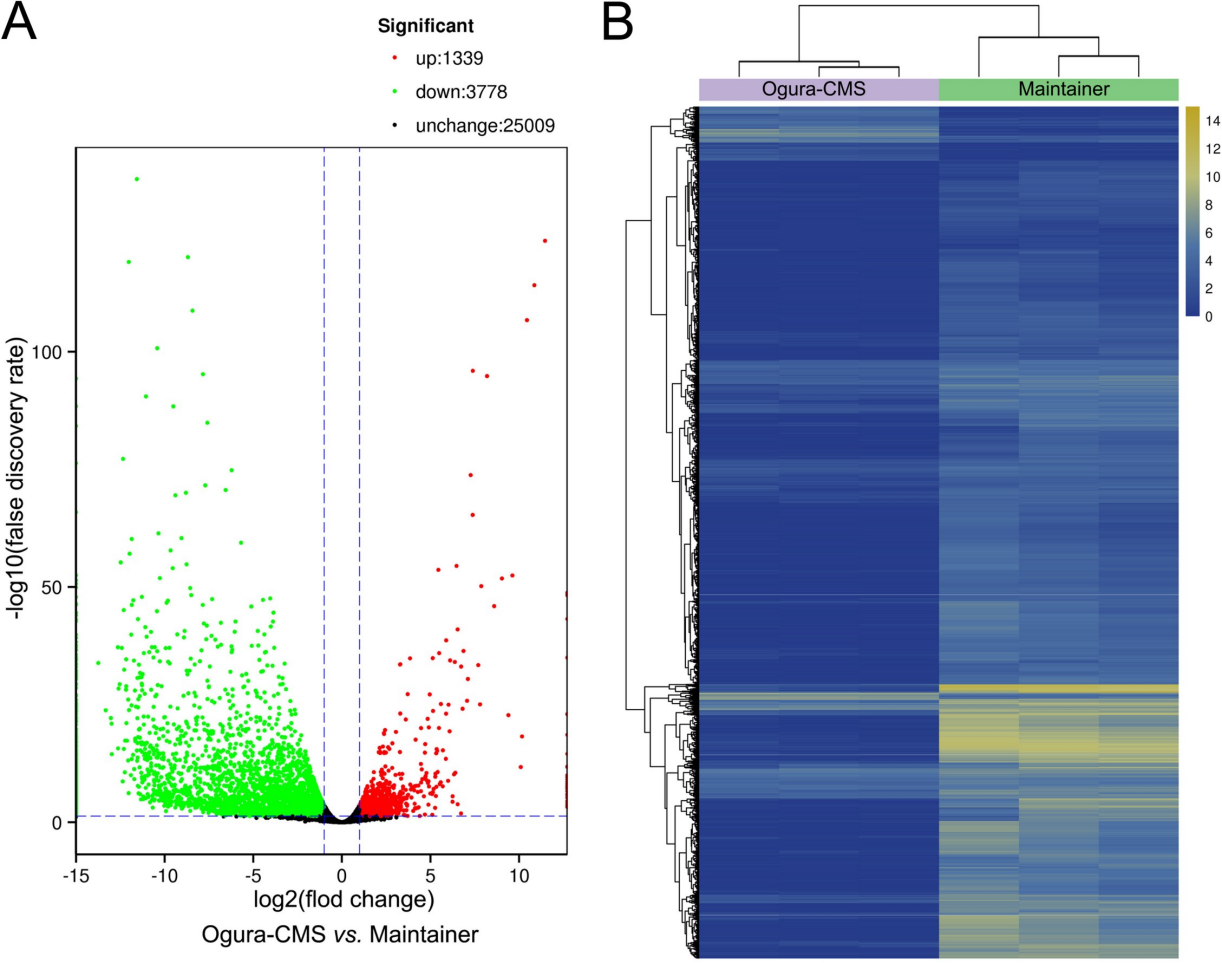
Expression profiles of differentially expressed genes (DEGs) in the Ogura-CMS and its maintainer fertile (MF) inflorescences of turnip. (A) Volcano plot showing significantly DEGs with log_2_ fold change (FC) ≥ 1 or ≤ -1 (Benjamini-Hochberg false discovery rate < 0.05). (B) A hierarchical clustering graph based on the expression values of all significantly DEGs identified in (A).

To determine whether the genes regulating pollen and tapetum development are defective or expressed abnormally, differential expression of genes during thereproductive development of the MF and CMS lines was analyzed. The reads were mapped to unigenes and quantified to show gene expression abundance by Fragments Per Kilobase of transcript per Million mapped reads (FPKM) [30]. The FPKM expression data were tested by correlation analysis to evaluate sampling between biological replicates, and all correlation coefficients were ≥ 0.82. Using the DESeq (2010) R Package with a FDR < 0.05 and a log_2_ FC ≥ 1 or ≤ -1, pairwise comparisons of Ogura-CMS *versus* MF showed that 5,117 genes were significantly differentially expressed, of which 1,339 genes were significantly up-regulated and 3,778 genes significantly down-regulated in the Ogura-CMS line relative to the MF line (Fig 7). Representative genes for the up- and down-regulated DEGs are listed in Tables 1 and 2, respectively, according to their functional categories. Referencing to the chromosome of Chinese cabbage (*Brassica rapa* ssp. *pekinensis*), the DEGs are widely distributed in all chromosomes (S4 Fig).

**Table 1:** Functional categories of representative genes significantly up-regulated in inflorescences of the Ogura-CMS line relative to its maintainer fertile (MF) line.

Gene ID	log_2_ fold change (Ogura-CMS line/MF line)	Description
**Transcription factors**		
TRINITY_DN24247_c4_g1	4.989846	Transcription factor MYB39
TRINITY_DN22677_c1_g9	3.653598	Probable WRKY transcription factor 71
TRINITY_DN25088_c3_g1	3.295980	NAC transcription factor 29
TRINITY_DN22677_c1_g2	3.196358	Probable WRKY transcription factor 71
TRINITY_DN23536_c1_g7	3.049643	Homeobox-leucine zipper protein ATHB-21
**Carbohydrate transport and metabolism**		
TRINITY_DN24721_c0_g1	5.481076	β-glucosidase 27
TRINITY_DN22492_c0_g5	5.408367	Peroxidase 15
TRINITY_DN24408_c2_g2	4.629681	Probable xyloglucan endotransglucosylase/hydrolase protein 18
TRINITY_DN22492_c0_g7	4.542994	Peroxidase
TRINITY_DN22492_c0_g3	3.409620	Peroxidase 49
**Lipid transport and metabolism**		
TRINITY_DN25317_c1_g5	1.323946	Fatty acyl-CoA reductase 1
TRINITY_DN24410_c0_g2	1.435586	Probable acyl-activating enzyme 16
TRINITY_DN25317_c1_g16	1.800464	Fatty acyl-CoA reductase 1
**Plant hormone signal transduction**		
TRINITY_DN22137_c0_g2	2.385851	Auxin-induced protein X15
TRINITY_DN24146_c0_g1	2.268715	Indole-3-acetic acid-amido synthetase GH3.5
TRINITY_DN24009_c3_g3	1.789990	Indole-3-acetic acid-induced protein ARG7
TRINITY_DN26266_c0_g2	1.751535	Serine/threonine-protein kinase SRK2J
TRINITY_DN23900_c1_g2	1.737397	Auxin-responsive protein IAA12

**Table 2:** Functional categories of representative genes significantly down-regulated in inflorescences of the Ogura-CMS line relative to its maintainer fertile (MF) line.

Gene ID	log_2_ fold change (Ogura-CMS line/MF line)	Description
**Transcription factors**		
TRINITY_DN25469_c0_g5	-8.944461	Zinc finger protein ZAT2
TRINITY_DN24004_c2_g7	-8.817322	NAC transcription factor 25
TRINITY_DN23291_c0_g9	-6.858249	Transcription factor GAMYB
TRINITY_DN25812_c2_g1	-6.813564	MADS-box transcription factor 16
TRINITY_DN22189_c1_g8	-6.333698	Probable WRKY transcription factor 31
**Carbohydrate transport and metabolism**		
TRINITY_DN27564_c2_g4	-12.994995	Exopolygalacturonase clone GBGA483
TRINITY_DN22409_c1_g6	-12.479006	Probable pectate lyase 4
TRINITY_DN26448_c4_g4	-11.859968	Pectinesterase 21
TRINITY_DN23204_c0_g1	-11.382811	Pectinesterase PPME1
TRINITY_DN27059_c3_g2	-11.281507	Xyloglucan endotransglucosylase/hydrolase protein 3
**Lipid transport and metabolism**		
TRINITY_DN24821_c1_g3	-9.749978	Delta(8)-fatty-acid desaturase 2
TRINITY_DN26166_c1_g2	-8.847981	Dehydrodolichyl diphosphate synthase 8
TRINITY_DN18964_c0_g2	-8.041336	Probable lipid phosphate phosphatase 4
TRINITY_DN22477_c0_g1	-7.414755	Long chain acyl-CoA synthetase 5
TRINITY_DN26830_c1_g1	-7.196643	Phosphoinositide phospholipase C 6
**Plant hormone signal transduction**		
TRINITY_DN22372_c0_g1	-9.961644	Indole-3-acetic acid-amido synthetase GH3.17
TRINITY_DN17602_c0_g1	-6.559842	4-substituted benzoates-glutamate ligase GH3.12
TRINITY_DN22992_c3_g4	-5.265387	Auxin-induced protein 15A
TRINITY_DN27694_c2_g5	-2.799438	ABSCISIC ACID-INSENSITIVE 5-like protein 1

### Functional annotation of differentially expressed genes during reproductive development of the Ogura-CMS line and its maintainer fertile line of turnip

**Fig 8:**
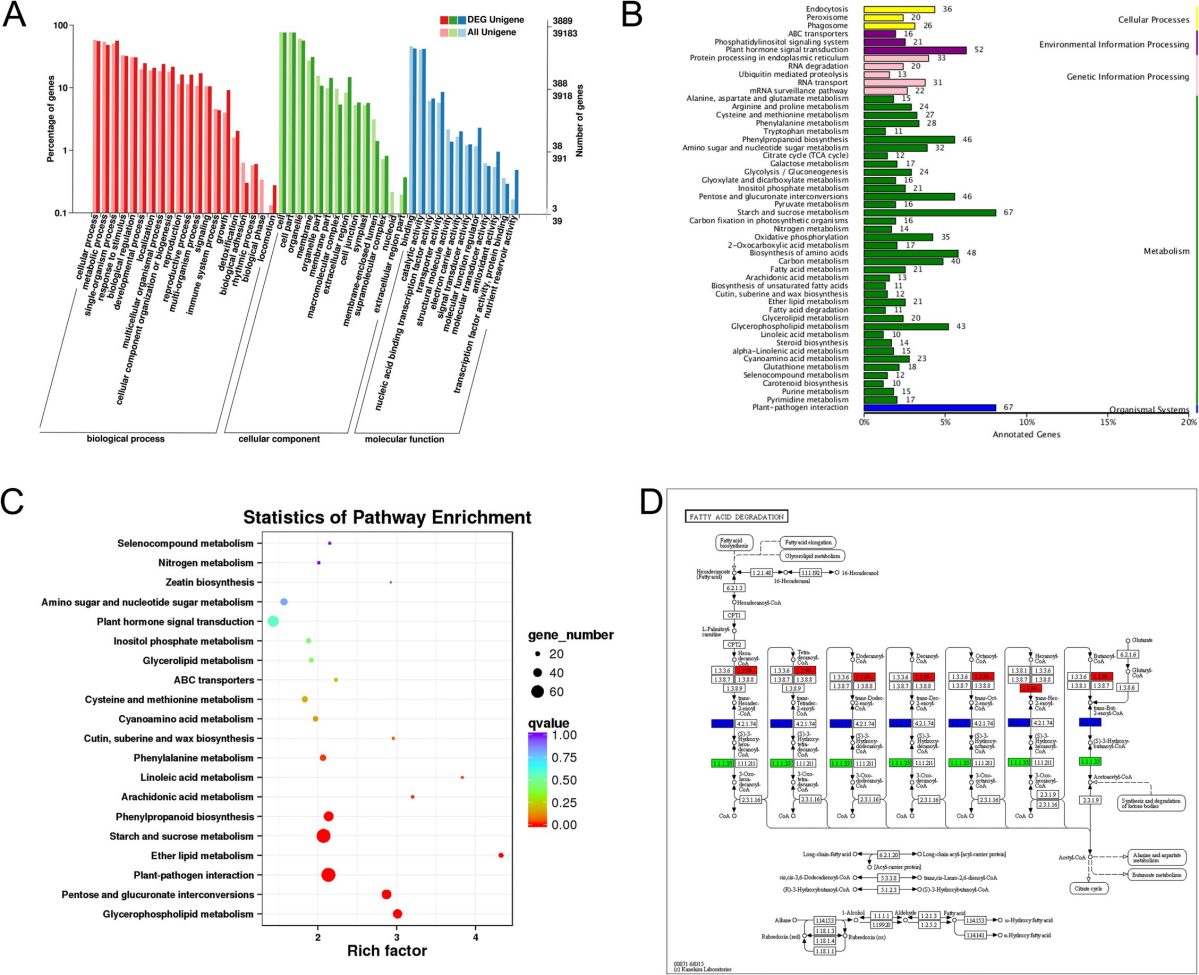
Gene Ontology (GO) and Kyoto Encyclopedia of Genes and Genomes (KEGG) pathway analyses. (A) GO annotations of all unigenes and differential expressed genes (DEGs) in the Ogura-CMS and its maintainer fertile (MF) inflorescences of turnip. The results are summarized in three main categories: biological process, cellular component and molecular function. The *y*-axis on the right indicates the number of genes in a category. The *x*-axis on the left indicates the percentage of a specific category of genes in that main category. (B) KEGG pathway annotations of DEGs. (C) KEGG pathway enrichment analysis of DEGs with top 20 enrichment scores. (D) KEGG pathway annotations of the fatty acid degradation pathway. Red marked nodes are associated with up-regulated genes; green nodes are associated with down-regulated genes; blue nodes are associated with both up-regulated and down-regulated genes.

To perform annotation analysis of the DEGs, eight public databases including COG, GO, KEGG, Swiss-prot database, KOG, Pfam, eggNOG, and Nr were searched. In total, 4,864 DEGs were found and annotated in detail in at least one of these databases (S4 Table). To forecast functional classifications of annotated DEGs, the GO and KEGG pathway analyses were performed to provide a clue. We utilized all DEGs for GO analysis and found that 76% of DEGs (3,889 out of 5,117) have at least one GO term assigned and were categorized into 46 functional groups (Fig 8A). Among these, the top three dominant categories were involved in cellular, metabolic, and single-organism processes (Fig 8A). The KEGG pathway analysis manifested that 50 pathways were significantly enriched, particularly metabolic pathways, plant-pathogen interaction, and plant hormone signal transduction (Fig 8B). The DEGs were found to be mostly enriched in ether lipid metabolism (Fig 8C). Furthermore, many genes involved in fatty acid metabolism which is essential for the assembly of exine and tryphine [3,31] were dysregulated (Fig 8D).

In all annotated DEGs, 1,289 genes were significantly up-regulated and 3,575 genes were significantly down-regulated in the Ogura-CMS relative to MF inflorescences (S4 Table). Among these, 610 DEGs were specifically expressed in the MF inflorescences and only 31 DEGs in the Ogura-CMS inflorescences (S5 and S6 Tables), implying that there is a considerable scope for further research to discover novel CMS-associated genes in turnip. In addition, several genes that were classified as function unknown but specifically expressed in the MF inflorescences, such as TRINITY_DN22922_c0_g2, TRINITY_DN37525_c0_g1, and TRINITY_DN23102_c0_g1, could be good candidates for CMS-related genes.

### Genes related to anther development and microspore formation

Based on genetic and transcriptomic studies, it has been long assumed that *Arabidopsis* pollen development involves precise spatial and temporal cooperation of the tapetum and the gametophyte itself, and relies on the functions of numerous genes and their dynamic regulatory network [32]. We compared the expressive alteration of homologs of *Arabidopsis* genes previously reported to be associated with anther and pollen development, to unravel whether cytoplasmic retro-regulated counterparts of those genes from the nucleus. In addition, some functionally known genes involved in this unique process in species other than *Arabidopsis* were also compared. As the innermost layer surrounding the sporogenous cells in the anther, the tapetum provides not only energy, but also nutrients, metabolites, and sporopollenin precursors for microspore development [33]. Coordinated with the defective tapetum, homologs of some extensively demonstrated genes and enzymes associated with tapetum development in *Arabidopsis* (Table 3) and other species (Table 4) exhibited altered expression in Ogura-CMS. For example, *AMS* is a basic helix-loop-helix (bHLH) transcription factor, one of the master regulators for tapetum and microspore development in *Arabidopsis* [3]. Expression of a counterpart of *AMS* (TRINITY_DN27860_c1_g1) was down-regulated in Ogura-CMS. Some turnip homologs of *AMS*-dependent genes including *QRT3* (TRINITY_DN22468_c2_g3), *CYP98A8* (TRINITY_DN26854_c1_g2), *CHS* (TRINITY_DN27063_c0_g1), *EXL6* (TRINITY_DN24807_c1_g1, and TRINITY_DN26730_c0_g3), and *PAB5* (TRINITY_DN22996_c2_g9, TRINITY_DN22996_c2_g5, and TRINITY_DN22996_c2_g10), showed reduced expression, but some genes did not, such as *CYP703A2*, *CYP704B1*, *KCS7*, *LAP5* and *LTP12* (Table 3). It was noteworthy that counterparts of two *AMS*-dependent genes, *TKPR1* (TRINITY_DN23571_c0_g2, TRINITY_DN25196_c0_g1, and TRINITY_DN23571_c0_g1) and *A6* (TRINITY_DN25127_c0_g3), suggested to be directly regulated by *AMS* in *Arabidopsis*, had increased expression in Ogura-CMS (Table 3). In addition, AMS regulatory pathway oriented analyses showed that turnip homologs of *ATA20* (TRINITY_DN25943_c1_g3 and TRINITY_DN25943_c1_g1), bHLH89 (TRINITY_DN24940_c2_g5), and *bHLH91* (TRINITY_DN22984_c1_g2 and TRINITY_DN22984_c1_g1) were down-regulated (Table 3), but *DYT1*, *TDF1*, *MS188*, and *MS1* were not in the pool of DEGs.

**Table 3:** List of known anther and microspore development-involved genes in *Arabidopsis* and turnip.

Gene ID	log_2_ fold change (Ogura-CMS line/ maintainer line)	Up/down-regulation (Ogura-CMS line/ maintainer line)	*Arabidopsis*			References
			Gene name	Locus	Description	
**Anther development/Tapetum development and degeneration**						
TRINITY_DN26683_c5_g2	1.652018	up	*RBOHE*	AT1G19230	NADPH oxidase	[34]
TRINITY_DN27860_c1_g1	-1.816880	down	*AMS*	AT2G16910	bHLH transcription factor	[33,35]
TRINITY_DN25433_c1_g3	1.150095	up	*BAM1*	AT5G65700	Leucine-rich repeat receptor-like serine/threonine-protein kinase	[36]
TRINITY_DN24940_c2_g5	-3.073631	down	*bHLH89*	AT1G06170	bHLH transcription factor	[37]
TRINITY_DN22984_c1_g2	-2.032122	down	*bHLH91*	AT2G31210	bHLH transcription factor	[37]
TRINITY_DN22984_c1_g1	-2.529790	down	*bHLH91*	AT2G31210	bHLH transcription factor	[37]
TRINITY_DN25943_c1_g3	-9.043751	down	*ATA20*	AT3G15400	Anther 20	[38]
TRINITY_DN25943_c1_g1	-9.481635	down	*ATA20*	AT3G15400	Anther 20	[38]
TRINITY_DN26731_c0_g2	-1.383573	down	*GPAT6*	AT2G38110	Glycerol-3-phosphate 2-O-acyltransferase	[39]
TRINITY_DN23446_c2_g8	-2.186224	down	*GPAT6*	AT2G38110	Glycerol-3-phosphate 2-O-acyltransferase	[39]
TRINITY_DN23672_c1_g1	-1.126727	down	*GPAT6*	AT2G38110	Glycerol-3-phosphate 2-O-acyltransferase	[39]
**Anther dehiscence**						
TRINITY_DN24200_c1_g2	-3.476626	down	*ADPG1*	AT3G57510	Polygalacturonase	[40]
TRINITY_DN24097_c1_g2	2.667609	up	*ADPG2*	AT2G41850	Polygalacturonase	[40]
TRINITY_DN24097_c1_g3	*	up	*ADPG2*	AT2G41850	Polygalacturonase	[40]
**Pollen development**						
TRINITY_DN25738_c0_g3	-1.398578	down	*ASKβ*	AT3G61160	Shaggy-like protein kinase	[41]
TRINITY_DN25738_c0_g5	-8.831513	down	*ASKβ*	AT3G61160	Shaggy-like protein kinase	[41]
TRINITY_DN25738_c0_g2	#	down	*ASKβ*	AT3G61160	Shaggy-like protein kinase	[41]
TRINITY_DN23175_c0_g2	-6.511601	down	*ASKβ*	AT3G61160	Shaggy-like protein kinase	[41]
TRINITY_DN25619_c0_g5	-2.895881	down	*AtMGT4/AtMRS2-3*	AT3G19640	Magnesium transporter	[42]
TRINITY_DN25619_c0_g7	-6.739006	down	*AtMGT9/AtMRS2-2*	AT5G64560	Magnesium transporter	[43]
TRINITY_DN23465_c1_g2	*	up	*Exportin-1*	AT5G17020	Exportin protein family protein	[44]
TRINITY_DN24192_c2_g4	#	down	*WRKY34*	AT4G26440	WRKY transcription factor	[45]
TRINITY_DN24036_c0_g3	-1.197370	down	*WRKY2*	AT5G56270	WRKY transcription factor	[45]
TRINITY_DN26176_c2_g4	-1.741099	down	*WRKY2*	AT5G56270	WRKY transcription factor	[45]
TRINITY_DN27988_c0_g1	-8.710511	down	*SCP/LBD27*	AT3G47870	LBD transcription factor	[46,47]
TRINITY_DN23313_c2_g1	-1.780221	down	*MYB32*	AT4G34990	R2R3 MYB transcription factor	[48]
TRINITY_DN23983_c0_g5	-1.147885	down	*MYB32*	AT4G34990	R2R3 MYB transcription factor	[48]
**Pollen wall development**						
TRINITY_DN23912_c2_g2	-4.032420	down	*CalS5*	AT2G13680	Callose synthase	[49]
TRINITY_DN24310_c1_g1	-6.224270	down	*CalS5*	AT2G13680	Callose synthase	[49]
TRINITY_DN24929_c2_g3	#	down	*ROCK1*	AT5G65000	Nucleotide sugar transporter	[50]
TRINITY_DN23139_c0_g6	-5.759143	down	*AtbZIP34*	AT2G42380	bZIP transcription factor	[51]
TRINITY_DN24551_c0_g3	2.504648	up	*CDM1*/*AtTTP*	AT1G68200	CCCH-type zinc finger protein	[52,53]
TRINITY_DN24329_c2_g4	-6.144312	down	*QRT1*	AT5G55590	Pectinesterase	[54,55]
TRINITY_DN24097_c1_g4	-4.462741	down	*QRT2*	AT3G07970	Polygalacturonase	[55]
TRINITY_DN22468_c2_g3	-4.018069	down	*QRT3*	AT4G20050	Polygalacturonase	[56]
TRINITY_DN22996_c2_g9	-5.452547	down	*PAB5*	AT1G71770	Polyadenylate-binding protein	[35]
TRINITY_DN22996_c2_g5	-4.602617	down	*PAB5*	AT1G71770	Polyadenylate-binding protein	[35]
TRINITY_DN22996_c2_g10	-5.410000	down	*PAB5*	AT1G71770	Polyadenylate-binding protein	[35]
TRINITY_DN24807_c1_g1	-8.742684	down	*EXL6*	AT1G75930	GDSL esterase/lipase	[33]
TRINITY_DN26730_c0_g3	-6.884745	down	*EXL6*	AT1G75930	GDSL esterase/lipase	[33]
TRINITY_DN27063_c0_g1	-7.907580	down	*CHS*	AT4G00040	Chalcone synthase	[35]
TRINITY_DN26854_c1_g2	-3.366574	down	*CYP98A8*	AT1G74540	Cytochrome P450	[33]
**Pollen exine development**						
TRINITY_DN23571_c0_g2	1.261739	up	*TKPR1*	AT4G35420	Tetraketide α-pyrone reductase 1	[57]
TRINITY_DN25196_c0_g1	2.205715	up	*TKPR1*	AT4G35420	Tetraketide α-pyrone reductase 1	[57]
TRINITY_DN23571_c0_g1	1.101840	up	*TKPR1*	AT4G35420	Tetraketide α-pyrone reductase 1	[57]
TRINITY_DN22625_c4_g1	-1.977965	down	*AtAPY6*	AT2G02970	ATP-diphosphohydrolase	[58]
TRINITY_DN24453_c1_g2	3.497293	up	*AtACBP4*	AT3G054420	Acyl-CoA-binding protein	[59,60]
**Pollen coat formation**						
TRINITY_DN27258_c1_g1	-1.035989	down	*FLP1/CER3*	AT5G57800	Aldehyde decarboxylase	[61]
TRINITY_DN25053_c4_g1	-6.822741	down	*ABCG9*	AT4G27420	ATP binding cassette transporter	[62]
TRINITY_DN27312_c0_g2	-4.364649	down	*ABCG31*	AT2G29940	ATP binding cassette transporter	[62]
TRINITY_DN27312_c0_g1	-3.151138	down	*ABCG31*	AT2G29940	ATP binding cassette transporter	[62]
TRINITY_DN22659_c0_g1	-11.567435	down	*GRP17*	AT5G07530	Oleosin-like protein	[63]
TRINITY_DN23861_c0_g2	-10.827264	down	*GRP17*	AT5G07530	Oleosin-like protein	[63]
TRINITY_DN22389_c1_g4	#	down	*GRP17*	AT5G07530	Oleosin-like protein	[63]
**Pollen intine development**						
TRINITY_DN25796_c0_g4	-11.203487	down	*FLA3*	AT2G24450	Fasciclin-like arabinogalactan protein	[64]
TRINITY_DN21829_c0_g1	-9.421529	down	*AGP6*	AT5G14380	Arabinogalactan protein	[65]
TRINITY_DN23900_c2_g6	#	down	*AGP11*	AT3G01700	Arabinogalactan protein	[65]
TRINITY_DN23900_c2_g1	-4.547789	down	*AGP11*	AT3G01700	Arabinogalactan protein	[65]
TRINITY_DN23148_c0_g1	-2.519718	down	*RPG1*	AT3G02230	Reversibly glycosylated polypeptide	[66]
TRINITY_DN27324_c0_g2	-1.867440	down	*RPG1*	AT3G02230	Reversibly glycosylated polypeptide	[66]
TRINITY_DN27324_c0_g1	-1.279723	down	*RPG2*	AT5G15650	Reversibly glycosylated polypeptide	[66]
TRINITY_DN23148_c0_g5	-2.965583	down	*RPG2*	AT5G15650	Reversibly glycosylated polypeptide	[66]
TRINITY_DN26054_c1_g12	1.360432	up	*ABCG1*	AT2G39350	ATP binding cassette transporter	[67]
TRINITY_DN24546_c0_g2	1.303258	up	*ABCG1*	AT2G39350	ATP binding cassette transporter	[67]

* specifically expressed genes in the Ogura-CMS inflorescences of turnip

# specifically expressed genes in inflorescences of the maintainer fertile (MF) line of turnip.

**Table 4:** List of known anther and microspore development-related genes in turnip and species other than *Arabidopsis*.

Gene ID	log_2_ fold change (Ogura-CMS line/ maintainer line)	Up/down-regulation (Ogura-CMS line/ maintainer line)	Species	Gene name	Description	References
**Anther development/Tapetum development and degeneration**						
TRINITY_DN23291_c0_g9	-6.858249	down	*Oryza sativa*	*GAMYB*	MYB transcription factor	[68,69]
TRINITY_DN24913_c2_g6	-3.344908	down	*Oryza sativa*	*GAMYB*	MYB transcription factor	[68,69]
TRINITY_DN24913_c2_g4	-5.085345	down	*Oryza sativa*	*GAMYB*	MYB transcription factor	[68,69]
**Pollen development**						
TRINITY_DN25127_c0_g3	1.157526	up	*Brassica napus*/*Oryza sativa*	*A6*/*Osg1*	Similar to β-1,3-glucanase	[70,71]
TRINITY_DN25738_c0_g3	-1.398578	down	*Brassica rapa*	*BrASK2*	Shaggy-like protein kinase	[72]
TRINITY_DN25738_c0_g5	-8.831513	down	*Brassica rapa*	*BrASK2*	Shaggy-like protein kinase	[72]
TRINITY_DN25738_c0_g2	#	down	*Brassica rapa*	*BrASK2*	Shaggy-like protein kinase	[72]
TRINITY_DN23175_c0_g2	-6.511601	down	*Brassica rapa*	*BrASK2*	Shaggy-like protein kinase	[72]
**Pollen wall development**						
TRINITY_DN27860_c1_g1	-1.816880	down	*Oryza sativa*	*TDR*	bHLH transcription factor	[73]
TRINITY_DN24360_c3_g3	-11.780927	down	*Brassica campestris*	*BcMF9*	Polygalacturonase 3 (PGA3)	[74]
TRINITY_DN26838_c0_g1	-11.405028	down	*Brassica campestris*	*BcMF9*	Polygalacturonase 3 (PGA3)	[74]
**Pollen intine development**						
TRINITY_DN27040_c1_g9	-9.032317	down	*Brassica campestris*	*BcPLL9*	Pectatelyase-like 9	[75]
TRINITY_DN27040_c1_g15	#	down	*Brassica campestris*	*BcPLL9*	Pectatelyase-like 9	[75]
TRINITY_DN27040_c1_g4	-8.186261	down	*Brassica campestris*	*BcPLL9*	Pectatelyase-like 9	[75]
TRINITY_DN27040_c1_g6	-9.846965	down	*Brassica campestris*	*BcPLL9*	Pectatelyase-like 9	[75]
TRINITY_DN23900_c2_g6	#	down	*Brassica campestris*	*BcMF8*	Arabinogalactan protein	[76,77]
TRINITY_DN23900_c2_g1	-4.547789	down	*Brassica campestris*	*BcMF8*	Arabinogalactan protein	[76,77]
TRINITY_DN21829_c0_g1	-9.421529	down	*Brassica campestris*	*BcMF18*	Arabinogalactan protein	[78]

# specifically expressed genes in inflorescences of the maintainer fertile (MF) line of turnip.

Based on our cytological results in pollen wall development of turnip, a dramatically altered expression of numerous counterparts of known genes associated with pollen wall formation in CMS florets (Tables 3 and 4) also seemed to be responsible for obvious defects in male gametophyte development. Accordingly, we suggest a somewhat similar regulatory network underlying microspore development in *Arabidopsis* and turnip.

Apart from the functionally known genes presented in Tables 3 and 4, 185 other novel DEGs were addressed to participate in male organ development in our GO analyses (S7 Table). Among these DEGs, 26 were specifically expressed in the MF inflorescences and none in the Ogura-CMS inflorescences (Table 5). Of the down-regulated genes, two were predicted to encode uncharacterized protein and four were functionally unknown, implying that they could be good candidates for further research to discover novel anther and microspore development-associated genes in turnip.

**Table 5:** Novel genotype-specific expressed genes with proposed function in male organ development based on GO analyses.

Gene ID	Maintainer line of turnip			Ogura-CMS line of turnip			Regulated	Proposed function
	Rep1_FPKM	Rep2_FPKM	Rep3_FPKM	Rep1_FPKM	Rep2_FPKM	Rep3_FPKM		
TRINITY_DN19577_c0_g2	1.25	1.08	1.12	0	0	0	down	Protein GAMETE EXPRESSED 2
TRINITY_DN23051_c0_g11	1.67	2.06	1.41	0	0.04	0	down	Zinc finger protein ZAT3
TRINITY_DN23051_c0_g7	1.67	2.06	1.41	0	0.04	0	down	Zinc finger protein ZAT3
TRINITY_DN21111_c0_g1	23.61	14.06	28.67	0	0	0	down	MADS-box transcription factor 56
TRINITY_DN27168_c1_g1	21.32	11.07	30.7	0	0	0	down	MADS-box transcription factor
TRINITY_DN26124_c2_g12	4.78	2.73	5.97	0	0	0	down	Transcription factor bHLH84
TRINITY_DN24536_c1_g10	13.25	5.04	49.07	0	0	0	down	Copper transporter 1-like
TRINITY_DN21496_c0_g1	4.45	9.51	3.35	0	0	0	down	Nicotianamine synthase
TRINITY_DN26355_c0_g14	75.57	24.19	181.55	0	0	0	down	ADP-ribosylation factor GTPase-activating protein AGD10
TRINITY_DN26355_c0_g13	61.77	24.55	145.28	0	0	0.17	down	ADP-ribosylation factor GTPase-activating protein AGD10
TRINITY_DN25568_c0_g4	0.68	1.2	0	0	0	0	down	Receptor-like protein 12
TRINITY_DN23305_c1_g3	14.47	5.96	22.17	0	0	0.1	down	E3 ubiquitin-protein ligase ORTHRUS 2
TRINITY_DN24462_c0_g13	23.71	15.5	22.11	0	0	0	down	Indole-3-acetic acid-amido synthetase GH3.9
TRINITY_DN22388_c0_g1	191.36	144.98	23.21	0	0	0	down	Non-specific lipid-transfer protein-like protein
TRINITY_DN22388_c0_g4	113.92	90.76	14.87	0	0	0.25	down	Non-specific lipid-transfer protein-like protein
TRINITY_DN22388_c0_g5	34.33	26.18	18.52	0	0	0	down	Non-specific lipid-transfer protein-like protein
TRINITY_DN26904_c2_g5	29.98	23.43	32.42	0	0	0	down	Probable methyltransferase PMT17
TRINITY_DN23083_c4_g4	37.56	23.91	15.94	0	0	0	down	Probable methyltransferase PMT17
TRINITY_DN26904_c2_g3	51.31	36.79	4.6	0	0	0	down	Probable methyltransferase PMT17
TRINITY_DN4311_c0_g1	2.76	1.4	1.71	0	0	0	down	Probable serine protease EDA2
TRINITY_DN24972_c0_g2	20.13	17.06	0.99	0	0	0	down	Uncharacterized protein
TRINITY_DN27618_c0_g1	78.27	95.23	75.59	0	0	0.69	down	Uncharacterized protein
TRINITY_DN22737_c1_g7	6.71	5.23	22.32	0	0	0	down	Function unkown
TRINITY_DN24700_c2_g1	35.39	23.24	13.14	0	0	0	down	Function unkown
TRINITY_DN22388_c0_g7	101.23	70.46	98.04	0	0	0	down	Function unkown
TRINITY_DN24377_c2_g5	6.15	8.72	19.02	0	0	0	down	Function unkown

Overall, these results indicated that mutation of cytoplasmic *ORF138* retro-regulated genes from the nucleus, and interactions between them led to male sterility in Ogura-CMS turnip.

## Discussion

### Morphological characteristics of Ogura-CMS turnip

Ogura-CMS originated from Japanese radish and has been extensively applied in hybrid breeding of crop species [1]. The morphological changes occurring during anther development, particularly in microspore and tapetum behavior, show considerable variation with different nuclear backgrounds and/or cytoplasmic genotype [8]. Here, we emphasized the value of detecting variation in apparently uniform turnip. In Ogura-CMS flowers of turnip, the length of the stamens was greatly reduced, with shortened filaments and thin withered white anthers (Figs 1 and 2). It has been proposed that filament elongation concerns the polar auxin transport from pollen grains/anther to filaments, and hormone-biosynthetic enzymes are also essential for stamen development, including filament elongation [79–81]. When RNA-Seq results were classified by function, we assumed that altered expression of the auxin-, jasmonate-, and gibberellin-related genes (Fig 8 and S4 Table) may be the reason for short Ogura-CMS filaments.

Further investigation showed that microspores exhibited aberrant development in Ogura-CMS anthers and this was initially observed at the uninucleate stage, leading to remnants of the aborted microspores with incomplete-developed layers of exine and tryphine (Fig 4). Defects in Ogura-CMS pollen wall formation were consistent with the observation that apart from the abortion of microspore development, the tapetum in Ogura-CMS anthers was swollen and abnormally vacuolated, initially at the tetrad stage (Figs 3 and 5). Tapetal organelles, such as elaioplasts and tapetosomes, were abnormally formed. In Brassicaceae plants, such as *Arabidopsis*, elaioplasts and tapetosomes are the carriers in the traffic of lipid molecules from the tapetum to the microspore [82]. Proper lipid accumulation in these organelles is critical for normal tapetum development and pollen wall formation, as disruptions of lipid metabolism-related genes affect tapetum morphology and pollen wall patterning [31]. These results showed that defective microspore production and early tapetum degeneration during microgametogenesis led to complete male sterility of the Ogura-CMS line.

It was also noteworthy that in sterile anthers, the middle layer persisted for a long time (Fig 5). The question is still poorly documented for that if other non-tapetal anther layers, such as the middle layer, endothecium, and epidermis, are also essential for microspore development. Previously, a delay in PCD of the middle layer was reported to induce male sterility in kiwifruit (*Actinidia deliciosa*) [83]. The absence of well-defined genes in which mutation leads to delayed degeneration of the middle layer has enriched our knowledge on microspore development. For instance, *Oryza sative tdr* mutant shows a delay in middle layer degeneration as well as tapetum PCD [73]. In our RNA-seq data, the turnip homologue (TRINITY_DN27860_c1_g1) of this bHLH transcription factor-encoding gene showed a significant decrease in transcript level in the Ogura-CMS line relative to the MF line (Table 4). Mutation of a leucine-rich repeat receptor protein kinase EXS/EMS1 in *Arabidopsis* causes abnormal persistence of the middle layer [84,85]. It is not clear what genes regulate the middle layer degeneration. Although non-tapetal anther layers argue against an essential role in pollen development, the molecular basis and effort of non-tapetal anther layers in microspore development still need further exploration in the future.

Morphology of all other floral organs was normal, suggesting that genes involved in floral organ identity were normal. Apart from affecting stamen development, several negative effects other than heterosis are resulted from the introduction of Ogura-CMS from radish, via *B*. *napus* into *B*. *rapa* [17]. It has been reported that the CMS mitochondrial signaling can also cause aberrations in other aspects including vegetative growth [2]. Though 10 generations of back-crossing, protoplast fusion eliminated the negative effects on crown diameter and plant height of Ogura-CMS plants, the fleshy root still had reduced size (Fig 1 and S2 Fig).

### Cytoplasmic retro-regulates nuclear gene expression

Based on findings on *Arabidopsis* microsporogenesis [86], the early stage of this process in the Ogura-CMS line should be normal (Fig 3). Instead, genes associated with tapetal development and meiotic tapetal function are defective in the Ogura-CMS line. Moreover, the expression of genes associated with microspore development could be altered. It has been reported in CMS rice, carrots, wheat, and *B*. *napus* that mitochondrial mutations affect nuclear gene expression, through a communication pathway from mitochondria to the nucleus called the mitochondrial retrograde signaling pathway [4–7]. In addition, variation in anther behavior among *Brassica* species suggests the presence of different regulatory mechanisms and/or multiple regulatory pathways [17]. Therefore, we explored the differential gene expression patterns of transcripts between the Ogura-CMS and its MF inflorescences in turnip. RNA-Seq identified prodigious DEGs (S4 Table), supporting an extensive involvement of mRNAs in anther development. The complex transcript network was simplified by modularization. A lower number of dysfunctional DEGs in the Ogura-CMS inflorescences may also reflect the underdevelopment of stamens. Overall, these results indicate that the regulatory network of anther development is altered in Ogura-CMS turnip, the nuclear genes involved in this complex process are retro-regulated by mitochondrial *orf138*, and mitochondrial-to-nucleus signaling in Ogura-CMS plants perhaps causes aberrant male sporophyte and gametophyte development.

### Genes involved in tapetum development and microspore formation show dysregulated expression

Pollen development involves delicate and coordinated spatio-temporal regulation of numerous biosynthetic genes by specific transcriptional regulators, and requires coordinated activity of different cell types and tissues of both gametophytic and sporophytic origin [3,32,87,88]. It is estimated that about 14,000 genes give a positive expression signal during microspore development, of which only a few have been shown to be pollen-specific with a defined requirement at different stages of pollen development [32]. In the CMS system, it may be more complicated because of the presence of the mitochondrial retrograde signaling pathway and the interaction between the nucleus and mitochondria [89]. We performed a transcriptomic comparison between the expression of some well-known anther and tapetum development-associated genes and their homologs in turnip. The results showed that cytoplasmic retro-regulated counterparts of some genes from the nucleus (Tables 3 and 4), such as *GPAT6*, which is essential for tapetum development, when disrupted causes defective exine deposition and partial pollen degradation [39]; *RBOHE*, encoding a NADPH oxidase and required for tapetal PCD, if interfered results in aborted male gametophytes [34]; and *BAM1*, a Leu-rich repeat receptor-like kinase encoding gene, is an important regulator for anther development [36].

During early tapetum development in *Arabidopsis*, *EXS*/*EMS1* is a trigger to the signaling pathway for tapetal fate determination [84,85]. Here, the counterparts of *EXS*/*EMS1* were not distinguishable in turnip (S3 and S4 Tables), indicating that retrograde signaling did not affect gene expression at the early stage of tapetum development and function, consistent with normal early tapetum behavior we observed (Fig 5). Later, core genetic networks involving several transcription factors regulate tapetum and microspore development, including a bHLH transcription factor *Arabidopsis AMS*/rice *TDR*, one of the master regulators [3]. *AMS* triggers at least 23 pollen wall development-related genes, such as *A6*, *TKPR1* [33]. Mutation in *AMS* causes tapetal hypertrophy extending into the locule and subsequently leads to abnormality of microspore development [33,35]. The turnip ortholog of *AMS* was found to be down-regulated in the Ogura-CMS inflorescence (Table 3). Some *AMS*-dependent genes were also dysregulated in the Ogura-CMS inflorescence, but others, such as *CYP703A2*, *CYP703B1*, *WBC27*, *TEK*, and *LAP5*, were not. In addition, a transcriptional regulatory pathway involving *AMS* with *DYT1*-*TDF1*-*AMS*-*MS188*-*MS1* has been proposed for tapetal and microspore development in *Arabidopsis* [3]. *DYT1* is a bHLH transcriptional factor and *TDF1* is a putative R2R3 MYB transcriptional factor, both of which are responsible for tapetum development and microspore formation [90,91]. However, the turnip orthologs of *DYT1* and *TDF1* were absent in our RNA-Seq data. Instead, the counterparts of the other two *Arabidopsis* bHLH transcriptional factors, *bHLH89* and *bHLH91* [37], and another R2R3 MYB transcription factor, *MYB32* [48], had decreased expression in the Ogura-CMS inflorescence. It has been suggested that *BrbHLH89* might replace *DYT1* function and transcription factor genes *BrTDF1*, *BrAMS*, *BrMS188*, and *BrMS1* in Chinese cabbage, however, it exhibited delayed or extended expression in the Ogura-CMS line [17]. Therefore, we suggest that the function of *AMS* may be retained but the regulatory *AMS* pathway may have some alteration between turnip and *Arabidopsis*, or other *Brassica* spp.

Recently, based on research on the inflorescence transcriptome of Ogura-CMS cabbage, 22 DEGs were assigned to pollen development [18]. Among these DEGs, some functionally known key members involving pollen development, such as *BoLAP5*, *BoLAP6*, *BoACOS5*, *BoMS2*, *BoCYP703A2*, and *BoCYP704B1*, were found to be down-regulated in Ogura-CMS anthers compared to the fertile line. In addition, some new members, such as *BobHLH1*, *BoMYB1*, and *BoMF2*, were also identified [92–94]. However, some of these well-defined genes were not part of the pool of our DEGs. These differences in gene expression may be the cause of different anther behaviors that Ogura-CMS cabbage has an abnormally fat tapetum with a delayed tapetum PCD, compared to our Ogura-CMS turnip which exhibited premature tapetum degeneration during microgametogenesis. These results provide new clues for a better understanding of different Ogura-CMS *Brassica* spp regulatory mechanisms.

Apart from those well-defined genes, 185 other novel DEGs were predicted to be involved in male organ development based on GO analyses, of which 26 DEGs were genotype-specific (S7 Table and Table 5), implying that they are good candidates for further research on novel anther and microspore development-associated genes in turnip. It is interesting that some members were previously reported to function in other developmental processes, not just anther development. For example, putative indole-3-acetic acid-amido synthetase GH3.9, a member of the GH3 family of auxin-responsive genes, was previously reported to contribute to primary root length [95]; *GAMETE EXPRESSED 2* encodes a trans-membrance domain containing protein which is targeted by *DUO1* in *Arabidopsis* [96]. These genes may be endowed with new putative roles in male organ development.

### Genes related to pollen wall development

The indispensable biological function of pollen is somehow reflected by its unique surrounding wall. Pollen wall synthesis is regulated by both the sporophytic tapetum and the microspore [97]. As the innermost layer of the pollen wall, intine not only plays an important role in aperture configuration and pollen stability, but also functions in pollen tube growth into the stigma [77,87,98,99]. Intine comprises of pectin, cellulose, hemicellulose, hydrolytic enzymes and hydrophobic proteins, and is controlled by the gametophyte [97,100]. Several genes and proteins have been identified in intine formation [66,74,75,97,101–107]. Here, we found that the expression of homologs of *RGP1*, *RGP2*, and *PLL9* in the Ogura-CMS inflorescences of turnip was reduced (Tables 3 and 4), disruptions of which contribute to the premature degradation of developing microspores [66,75]. Arabinogalactan proteins (AGPs) are extensively glycosylated hydroxyproline-rich glycoproteins and are also proved to be indispensable for intine formation [78,108]. Currently, two classical AGP-encoding genes *AGP6* and *AGP11* and, their *B*. *campestris* ortholog *BcMF18* and *BcMF8*, and a fasciclin-like AGP (FLA) gene *FLA3*, have been isolated and identified [77,78,109,110]. The orthologs of all these AGP-encoding genes were found in turnip and showed down-regulated expression in the Ogura-CMS inflorescences (Tables 3 and 4). In addition, counterparts of another two FLA genes, *FLA5* (TRINITY_DN22476_c2_g15 and TRINITY_DN22476_c2_g16) and *FLA14* (TRINITY_DN23986_c3_g2, TRINITY_DN23986_c3_g5, and TRINITY_DN23986_c3_g6), showed down-regulation in the Ogura-CMS inflorescences (S4 Table). Similarly, in Chinese cabbage, *BrFLA14* was highly expressed in fertile buds and could be associated with pollen development [72], implying that this FLA gene could be important for intine formation in turnip.

In addition to the extensively reviewed genes and enzymes associated with pollen intine formation summarized above, the expression of some genes acting at different steps of exine formation was also dysregulated (Table 3). Exine formation requires proper pollen wall pattern determination and sporopollenin deposition. Pollen wall pattern determination is dependent on the formation and dissolution of the callose wall, which provides a structural basis for the primexine deposition [3]. Previous attempts to explore the roles of callose wall-related genes have indicated that overexpression of β-1,3-glucanase in the tapetum results in defective exine pattern formation [111]. *Brassica napus* and *Arabidopsis A6* and its rice ortholog *Osg1* encode β-1,3-glucanase that are essential for timely callose degradation [70,71]. As for the abnormality of tapetum, their counterpart in turnip (TRINITY_DN25127_c0_g3) was found to be up-regulated in the Ogura-CMS inflorescences (Table 4). In addition, the counterparts of *CalS5* (TRINITY_DN23912_c2_g2 and TRINITY_DN24310_c1_g1), in which mutation causes abnormal exine wall formation [49], had a decreased expression in the Ogura-CMS inflorescences (Table 3). Although the appearance of the Ogura-CMS callose wall at the tetrad stage was normal, the dysregulated expression of homologous genes of *A6*/*Osg1* and *CalS5* was suggested to be positively correlated with efficient microspore development. Sporopollenin deposition is dependent on the synthesis, secretion and translocation of sporopollenin precursors, which is closely associated with fatty acid metabolism [3,31]. In our RNA-Seq data, many genes involved in fatty acid metabolism pathway were dysregulated in the Ogura-CMS inflorescences (Fig 8D), indicating that they might also be responsible for exine defects. Notably, a number of functionally known key members of the complex biochemical biosynthesis of sporopollenin precursors in the tapetum were not in the pool of DEGs. ACOS5, a medium- and long-chain fatty acyl CoA-synthetase [112], are condensed with fatty acyl ACP reductase MS2 [113], cytochrome P450 family members CYP703As and CYP704Bs [114–116], flavonoid synthases LAP5 (PKSA) and LAP6 (PKSB) [117], together with tetraketide α-pyrone reductases TKPR1 and TKPR2 [57], yield precursors of sporopollenin building units. Disruption of these genes drastically hampers biosynthesis of sporopollenin precursors in the tapetum. Among all these genes, only the counterparts of *TKPR1* were found up-regulated in our RNA-Seq results. We suggest that the reduced expression of ATP-binding cassette subfamily G (ABCG) transporters contributes to the transport of sporopollenin precursors across anther tissues [67]. Here, we only found *ABCG1* had decreased expressed counterparts in the Ogura-CMS inflorescences (Table 3), which functions redundantly with *ABCG16* in exine layer formation. These results suggest that the synthesis and secretion of sporopollenin precursors may be little affected by mitochondrial retrograde signaling. In addition, RNA-Seq analyses showed that a large number of lipid transfer proteins (LTPs)-encoding genes were dysregulated (S4 Table). Presently, no experimental evidence was obtained for the mechanism by which LTPs sporopollenin precursors are translocated, but LTPs are likely candidates for their delivery from the tapetum to the microspores [100]. Overall, these results indicated that during the process of sporopollenin deposition in Ogura-CMS turnip, the focal point of the effect of mitochondrial *orf138* may be sporopollenin precursor translocation.

Genes related to pollen coat formation, which is also closely associated with fatty acid metabolism, are also indispensable in Ougra-CMS turnip. *FLP1*/*CER3*, is a member of the ECERIFERUM family, which appears to participate in the synthesis of wax, components of tryphine and sporopollenin of exine in *Arabidopsis* [61]. In the *flp-1 mutant*, excessive tryphine fills the cavity of the exine leading to a smooth surface that is sensitive to acetolysis. We found the homolog of *FLP1*/*CER3* (TRINITY_DN27258_c1_g1) was down-regulated (Table 3). In addition, the expression of the counterparts of *ABCG9* (TRINITY_DN25053_c4_g1), *ABCG31* (TRINITY_DN27312_c0_g2 and TRINITY_DN27312_c0_g1) and *GRP17* (TRINITY_DN22659_c0_g1, TRINITY_DN23861_c0_g2, and TRINITY_DN22389_c1_g4), which contribute to the accumulation of pollen coat [62,63], was also decreased (Table 3). The altered expression of genes associated with the pollen coat may be related to male sterility in Ogura-CMS turnip.

## Conclusions

In this study, detailed morphological characteristics of an Ogura-CMS line and its MF line of turnip were described. We employed high-throughput sequencing approaches to identify candidate nuclear genes that responded to mitochondrial retrograde signaling, and focused on the anther and microspore development-associated genes. Comparison of the gene expression between CMS and MF lines supported the notion that CMS is attributed to the mitochondrial retrograde signaling pathway and the interaction of nuclear and organelle genomes. Furthermore, our results suggest that different regulatory mechanisms and/or multiple regulatory pathways may exist in *Brassica* spp.

## Supporting information

S1 Fig Observation of floral organs from the Ogura-CMS line and its maintainer fertile (MF) line at anthesis stage of turnip. (A-D) Scanning electron microscopy observation of floral organ morphology in the MF line. (E-H) Scanning electron microscopy observation of floral organs in the Ogura-CMS line showing normal morphology that are similar to those of the MF line. (A, E) The outer surface of a petal. (B, F) The outer surface of a sepal with numerous stomata (indicated by arrows). (C, G) The finger-like papillae of a mature stigma with a smooth surface. Arrows indicate the stomata on the style outer epidermis. (D, H) The outer surface of a nectary. Bars = 50 μm in (A, E), 100 μm in (B, F), 300 μm in (C, G), 200 μm in (D, H).
(JPG)

S2 Fig Phenotypic comparison of the Ogura-CMS line and its maintainer fertile (MF) line of turnip. (A) External morphology of seedlings at 32 days after germination. (B) The fleshy roots thickening at 48 days after germination. (C) Morphological features of flowering plants at 180 days after germination. (D) The length and diameter of fleshy roots at 180 days after germination. The values are the mean ± SD (standard deviation). (E) Normal silique growth and seed set on an Ogura-CMS plant and a MF plant pollinated with MF pollen grains as the male donor. Bars = 5 cm.
(JPG)

S3 Fig Statistics of *de novo* assembly of transcriptome in the Ogura-CMS and its maintainer fertile (MF) inflorescences of turnip. (JPG)

S4 Fig Differential expressed genes (DEGs) in the Ogura-CMS and its maintainer fertile (MF) inflorescences of turnip on Chinese cabbage chromosomes. From the outside in, the first circle of the Circos plot is a chromosome map of the Chinese cabbage genome. The homologous genes of all DEGs of turnip in Chinese cabbage are showed in the second circle. All DEGs are marked in the third circle.
(JPG)

S1 Table List of primers used in real-time RT-PCR validation in turnip. (XLSX)

S2 Table Numbers of RNA-Seq reads for inflorescences from the Ogura-CMS line and its maintainer fertile (MF) line of turnip. (XLSX)

S3 Table The RNA-Seq data obtained in the *de novo* assembly from inflorescences of the Ogura-CMS line and its maintainer fertile (MF) line of turnip. (XLSX)

S4 Table Detailed annotation information of differential expressed genes (DEGs) in the Ogura-CMS and its maintainer fertile (MF) inflorescences of turnip. (XLSX)

S5 Table List of specifically expressed genes in inflorescences of the maintainer fertile (MF) line of turnip. (XLSX)

S6 Table List of specifically expressed genes in the Ogura-CMS inflorescences of turnip. (XLSX)

S7 Table Novel genes with putative function in male organ development based on GO analyses. (XLSX)

## Abbreviations

ABCG: ATP-binding cassette subfamily G
AGP: arabinogalactan proteins
bHLH: basic helix-loop-helix
CMS: cytoplasmic male sterility
COG: Clusters of Orthologous Group
DEG: differentially expressed gene
eggNOG: orthologous groups of genes
FC: fold change
FDR: false discovery rate
FLA: fasciclin-like AGP
FPKM: Fragments Per Kilobase of transcript per Million mapped reads
GO: Gene ontology
KEGG: Kyoto Encyclopedia of Genes and Genomes
KOG: Clusters of Protein homology database
LTP: lipid transfer protein
MF: maintainer fertile
NCBI: National Center for Biotechnology Information
Nr: NCBI non-redundant protein
PCD: programmed cell death
Pfam: Homologous protein family
PPI: predicted protein-protein interaction
RNA-seq: RNA sequencing
RT-PCR: reverse transcription polymerase chain reaction
STRING: Search Tool for the Retrieval of Interacting Genes/Proteins
TEM: transmission electron microscopy.
