# Supplementary figures and images for: Comprehensive analysis of Ogura cytoplasmic male sterility-related genes in turnip (Brassica rapa ssp. rapifera) using RNA sequencing analysis and bioinformatics
Source: PLoS One. 2019 Jun 14;14(6):e0218029. doi: 10.1371/journal.pone.0218029 (PMC6568414; doi:10.1371/journal.pone.0218029)

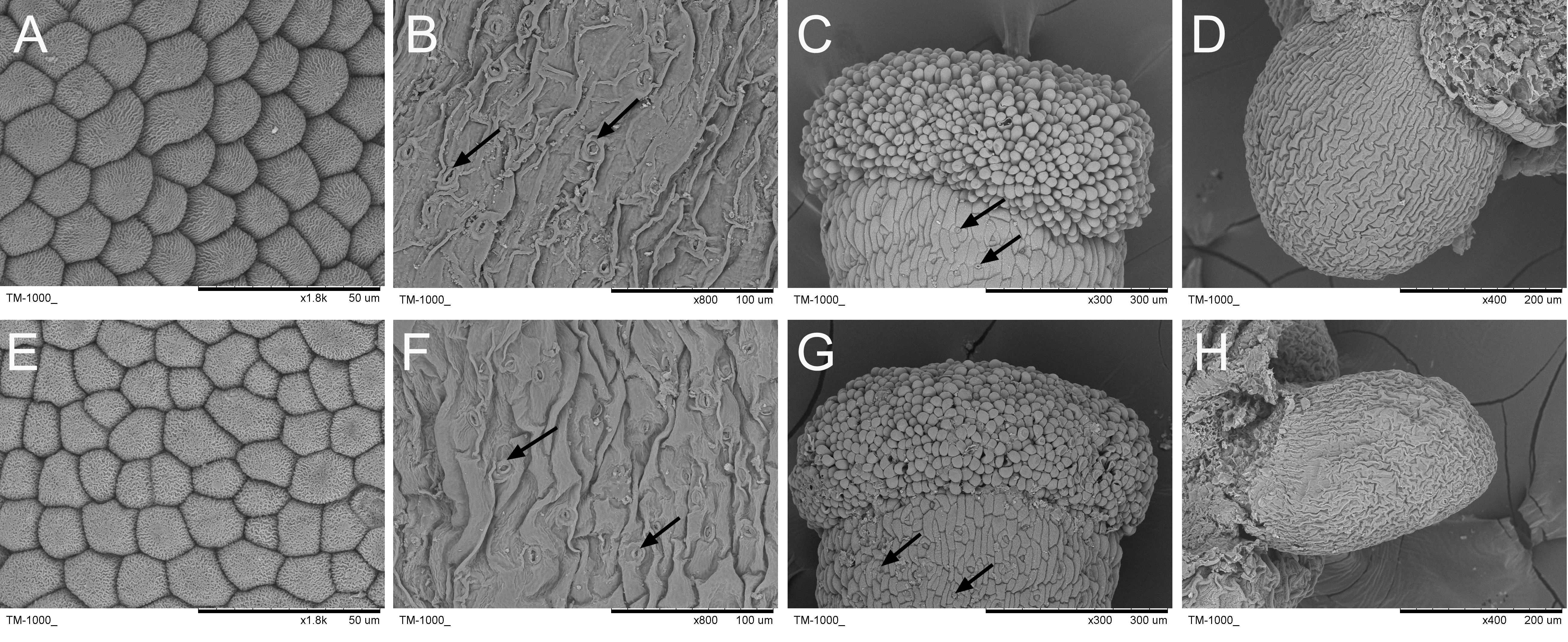

Supplement: S1 Fig — (A-D) Scanning electron microscopy observation of floral organ morphology in the MF line. (E-H) Scanning electron microscopy observation of floral organs in the Ogura-CMS line showing normal morphology that are similar to those of the MF line. (A, E) The outer surface of a petal. (B, F) The outer surface of a sepal with numerous stomata (indicated by arrows). (C, G) The finger-like papillae of a mature stigma with a smooth surface. Arrows indicate the stomata on the style outer epidermis. (D, H) The outer surface of a nectary. Bars = 50 μm in (A, E), 100 μm in (B, F), 300 μm in (C, G), 200 μm in (D, H). (JPG) [file pone.0218029.s001.jpg]

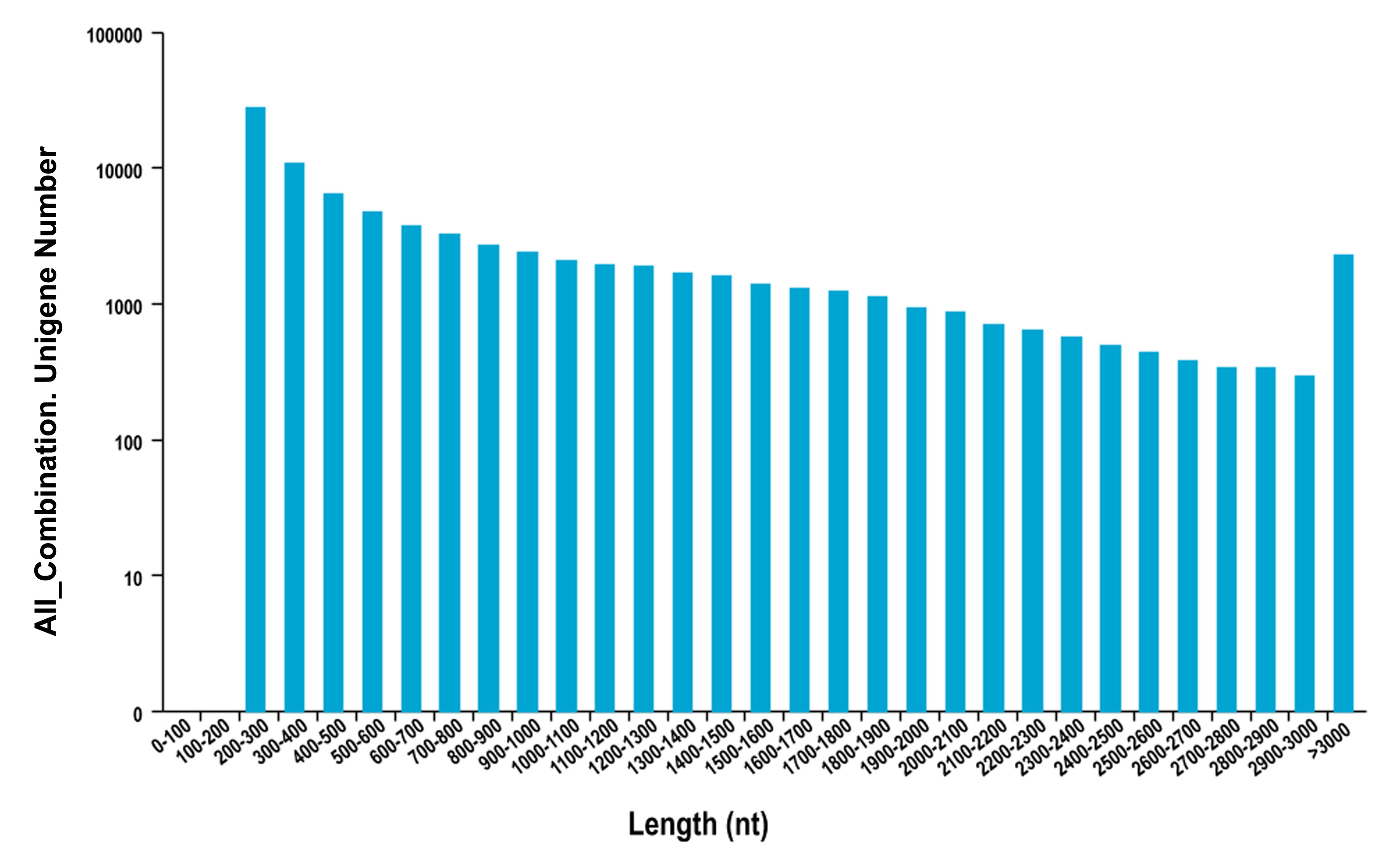

Supplement: S3 Fig — (JPG) [file pone.0218029.s003.jpg]

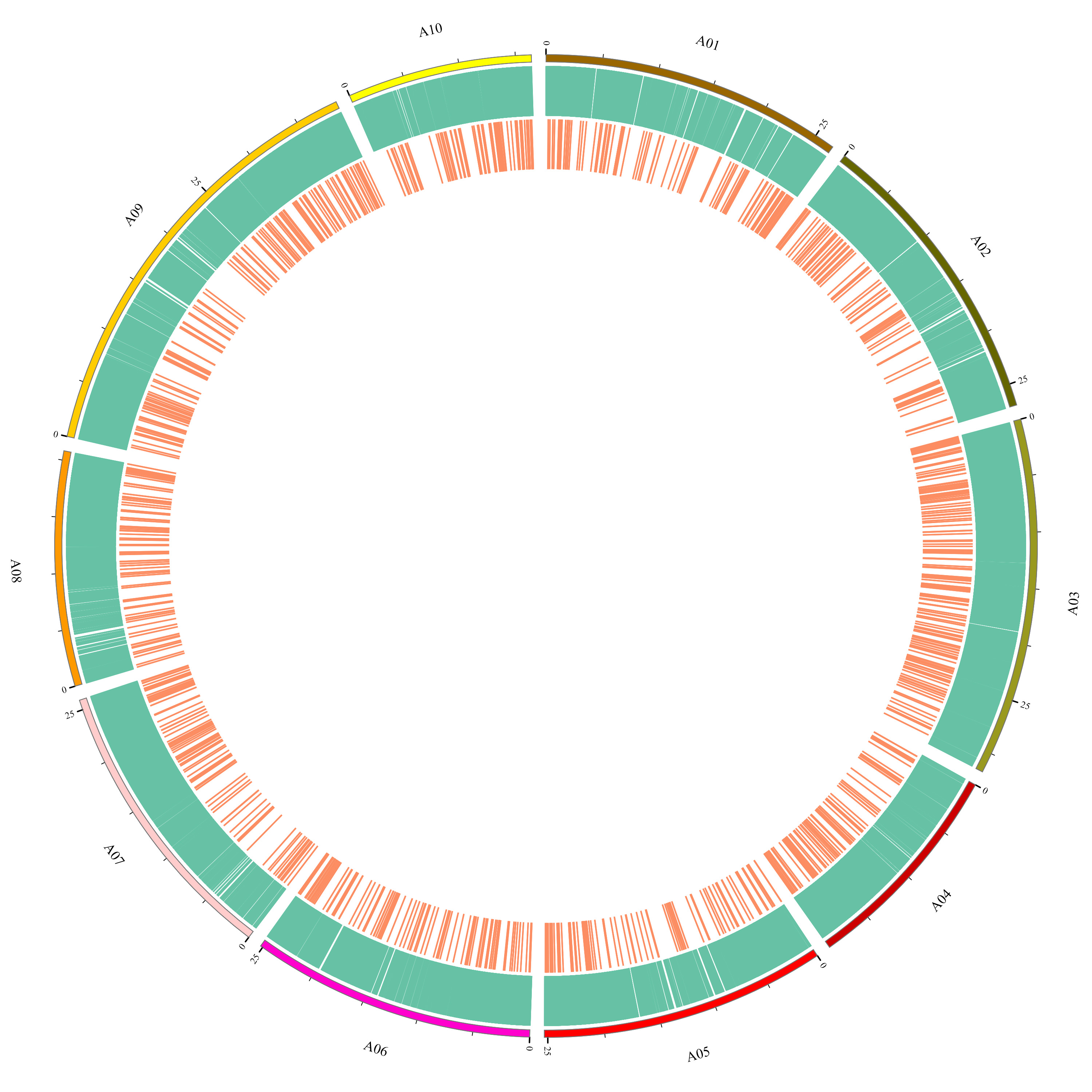

Supplement: S4 Fig — From the outside in, the first circle of the Circos plot is a chromosome map of the Chinese cabbage genome. The homologous genes of all DEGs of turnip in Chinese cabbage are showed in the second circle. All DEGs are marked in the third circle. (JPG) [file pone.0218029.s004.jpg]
